# Fructose-bisphosphatase1 (FBP1) alleviates experimental osteoarthritis by regulating Protein crumbs homolog 3 (CRB3)

**DOI:** 10.1186/s13075-023-03221-5

**Published:** 2023-12-04

**Authors:** Zhuolun Wang, Xinjie Wang, Liangliang Liu, Xiongtian Guo, Haiyan Zhang, Jianbing Yin, Rengui Lin, Yan Shao, Daozhang Cai

**Affiliations:** 1 Department of Orthopedics, Orthopedic Hospital of Guangdong Province, Academy of Orthopedics·Guangdong Province, Guangdong Provincial Key Laboratory of Bone and Joint Degeneration Diseases, The Third Affiliated Hospital of Southern Medical University, Guangzhou, 510630 Guangdong China; 2 Department of Joint Surgery, Center for Orthopedic Surgery, Orthopedic Hospital of Guangdong Province, The Third School of Clinical Medicine, Southern Medical University, The Third Affiliated Hospital of Southern Medical University, Guangzhou, Guangdong China

**Keywords:** Osteoarthritis, Cartilage, Gluconeogenesis, Chondrocyte, Senescence

## Abstract

**Purpose:**

To identify the role of gluconeogenesis in chondrocytes in osteoarthritis (OA).

**Materials and methods:**

Cartilage samples were collected from OA patients and C57 mice and were stained with Safranin O-Fast Green to determine the severity of OA. Periodic acid Schiff staining was used to characterize the contents of polysaccharides and SA-βGal staining was used to characterize the aging of chondrocytes. Immunohistochemistry and western blotting were used to detect fructose-bisphosphatase1 (FBP1), SOX9, MMP13, P21, and P16 in cartilage or chondrocyte. The mRNA levels of fbp1, mmp13, sox9, colX, and acan were analyzed by qPCR to evaluate the role of FBP1 in chondrocytes.

**Results:**

The level of polysaccharides in cartilage was reduced in OA and the expression of FBP1 was also reduced. We treated the chondrocytes with IL-1β to cause OA in vitro, and then made chondrocytes overexpress FBP1 with plasma. It shows that FBP1 alleviated the degeneration and senescence of chondrocytes in vitro and that it also showed the same effects in vivo experiments. To further understand the mechanism of FBP1, we screened the downstream protein of FBP1 and found that CRB3 was significantly downregulated. And we confirmed that CRB3 suppressed the degeneration and delayed senescence of chondrocytes.

**Conclusions:**

FBP1 promoted the polysaccharide synthesis in cartilage and alleviated the degeneration of cartilage by regulating CRB3, so FBP1 is a potential target in treating OA.

**Supplementary Information:**

The online version contains supplementary material available at 10.1186/s13075-023-03221-5.

## Introduction

Osteoarthritis (OA) is a chronic joint disease characterized by cartilage destruction and osteophyte hyperplasia, which causes joint pain and dysfunction, and is a major cause of disability [1–4]. OA, as a relatively common chronic disease, causes extensive worldwide economic loss every year and is a serious burden to many families [5, 6]. At present, the non-operation treatment of OA has been largely limited to steroidal or nonsteroidal anti‐inflammatory drugs that provide symptomatic relief from pain and inflammation [7]. The main pathological change in OA is the degeneration of cartilage, so studying the mechanism of cartilage degeneration and damage is important in understanding and treating OA. Regarding the mechanism of OA, studies reported that in the extracellular matrix, which is the main component of cartilage, the main reason for the destruction of OA cartilage was an imbalance between its synthesis and decomposition, [8, 9] and the extracellular matrix (ECM) not only has the role of carrying weight and protecting chondrocytes, but it also regulates cellular activity and plays an important role in the metabolism of cartilage [10]. During OA, a large number of factors such as matrix metalloproteinase (MMP13) are present in cartilage tissue, which promote the destruction of cartilage ECM and accelerate the progression of OA [11, 12]. In addition, cellular senescence also promotes the development of OA, which will cause chondrocytes to produce substances such as IL-1β and IL-6, to aggravate joint inflammation and affect cell metabolism, resulting in further progression of OA [13–15].

The progression of OA is associated with many risk factors, including obesity, genetics, and lifestyle, with the most important risk factor being aging [16]. In cartilage, the senescence of chondrocytes is considered as an important phenotype for the development of OA [17]. Metabolic changes and decreased cell division during senescence lead to elevated lysosomes in cells, so cellular senescence can be monitored by measuring galactosidase β activity [18, 19]. P21 and P16 are markers of cellular senescence; the elevation of P21 is closely related to damage of cellular DNA, and its deletion can promote the proliferation of chondrocytes and repair of cartilage [20–22]. Although the expression of P16 in OA chondrocytes significantly increases, its contribution to the development of OA has not yet been determined [23].

Carbohydrate metabolism is the most important metabolic process of the human body, which provides energy for the body and raw materials for the synthesis of glycans and other substances. In cartilage, sugar metabolism acts as the most important means of energy metabolism of chondrocytes, and also participates in the synthesis of glycosaminoglycans and other substances, which comprise the main matrix of cartilage, and which play a vital role in the production and repair of cartilage [12, 24]. There is growing evidence that the incidence and severity of OA increase with diabetes, which is related to systemic glucose metabolism in joints, and that there is a strong link between glucose metabolism in chondrocytes and the pathogenesis of OA [25–27]. However, as one of the main components of the extracellular matrix, the synthesis of glycosaminoglycans requires the gluconeogenesis pathway to provide a large amount of glucose as raw material, so studying the influence of gluconeogenesis on OA is important [28]. Fructose-bisphosphatase 1 (FBP1) is a key enzyme in the gluconeogenesis pathway, which is expressed in most cells, plays a key role in regulating gluconeogenesis, and affects the synthesis of glycogen and other polysaccharide substances [29]. However, the relationship between FBP1 and OA is unclear.

In this study, we investigated changes in gluconeogenesis and how FBP1 expression is decreased during OA. We hypothesized that FBP1 played an important role in influencing OA progression. To test this hypothesis, we conducted in vivo and in vitro experiments to confirm the protective effect of FBP1 on articular cartilage, and studied its downstream proteins to further elucidate its mechanism of action.

## Results

### Loss of FBP1-expressing chondrocytes in patients with OA and in OA mice

**Fig. 1:**
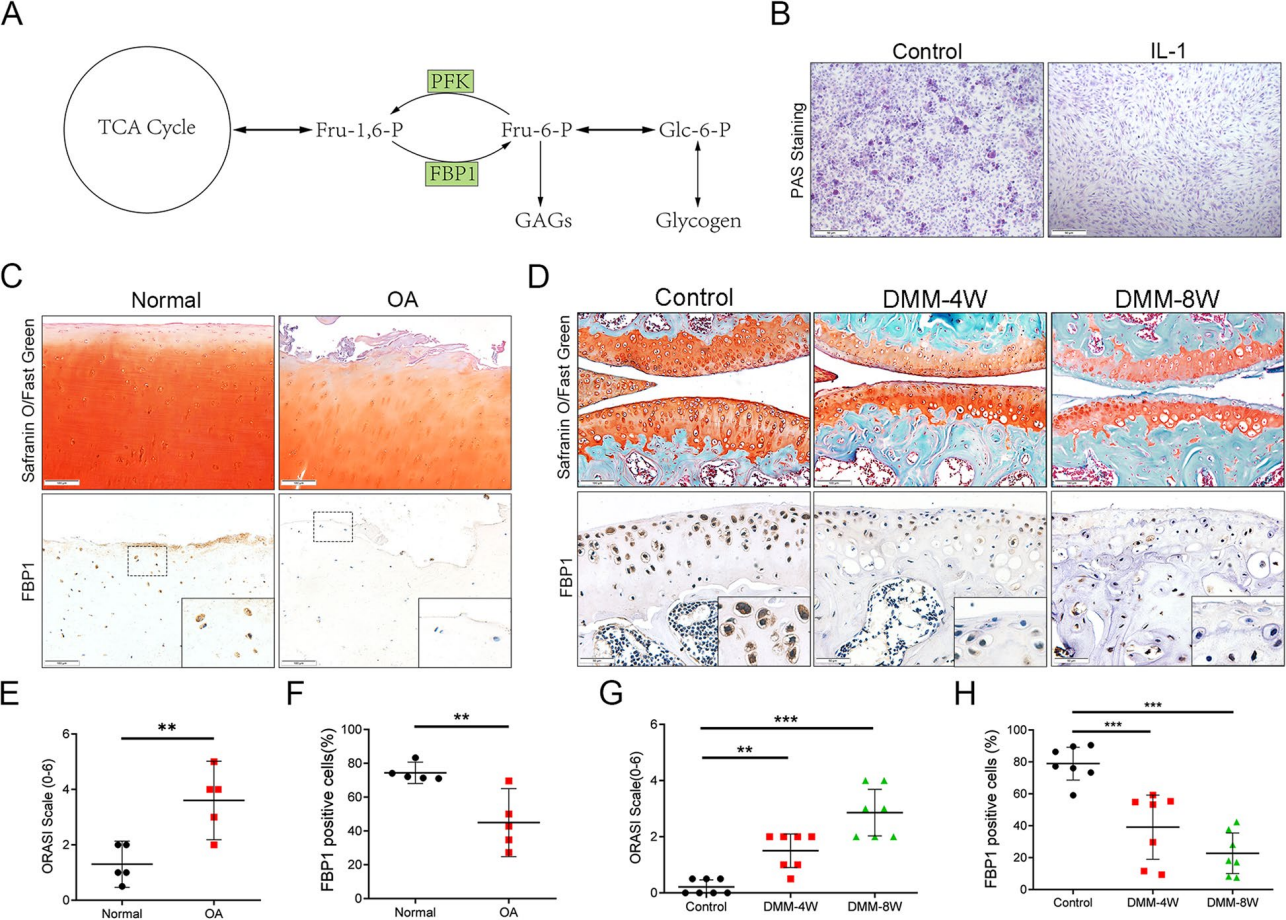
Loss of FBP1-expressing chondrocytes in patients with OA and in OA mice. **A** The schematic diagram of the process of gluconeogenesis. **B** The PAS staining of the primary chondrocytes treated with IL-1. Scale bars = 50 μm. **C** The Safranin O and fast green staining of human cartilage of OA patients and immunohistochemistry staining for FBP1. Scale bars = 100 μm. **D** Safranin O and Fast Green staining of controls and destabilization of medial meniscus (DMM) knee cartilage of C57 mouse, and FBP1 levels detected by immunohistochemistry staining. DMM-4W means tissues obtained 4 weeks after the DMM surgery, and DMM-8W means tissues obtained 8 weeks after the DMM surgery. Scale bars = 100 μm. Scale bars = 50 μm. Quantitative analysis of the OARSI scale and FBP1-positive chondrocytes in OA patients (**E, F**) or in DMM mice (**G, H**). *n* = 5 per group in human. *n* = 7 per group in mice. *p < 0.05, **p < 0.01, ***p < 0.001, NS = not significant. One-way analysis of variance (ANOVA) was performed

To confirm whether the carbohydrate metabolism of chondrocytes was altered during OA, we stimulated the primary chondrocytes of mice with IL-1, and then determined possible changes of polysaccharides. The results of periodic acid Schiff (PAS) staining showed that polysaccharides like glycogen were reduced after stimulation with IL-1, which showed that gluconeogenesis was inhibited (Fig. 1B). Cartilage, which consists of chondrocytes and extracellular matrix-like aggrecan and collagen II, needs large amounts of glucose to synthesize aggrecan. As an important process providing sufficient amounts of glucose, gluconeogenesis therefore plays an important role in the synthesis of cartilage (Fig. 1A).

To confirm the expression of FBP1 in OA cartilage, we used OA cartilage from humans and mice to conduct immunohistochemical staining for FBP1 (Fig. 1C–H). The results showed that compared with the control group, the expression of FBP1 in cartilage of the OA group from humans and mice significantly decreased.

### FBP1 restores the metabolic disorders of chondrocytes and delays its aging in OA

**Fig. 2:**
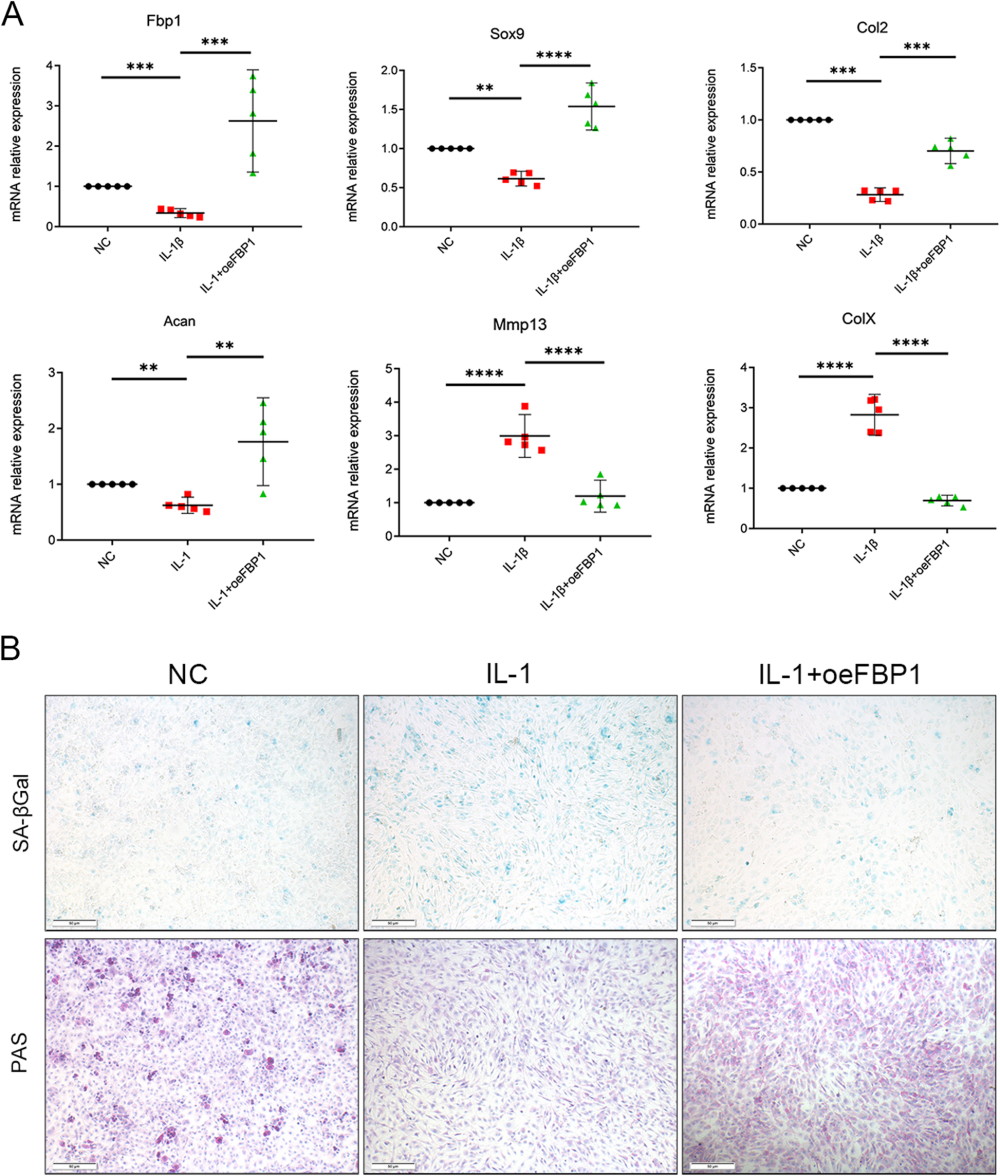
FBP1 restores the metabolic disorders of chondrocytes and delays its aging in OA. Mouse primary chondrocytes were stimulated with IL-1β for 24 h and then treated with or without granules that could overexpress FBP1. **A** Quantitative PCR analysis of FBP1, SOX9, COL2A1, ACAN, MMP13, and ColX in chondrocytes. *n* = 5 per time point. *p < 0.05, **p < 0.01, ***p < 0.001, NS = not significant. One-way analysis of variance (ANOVA) was performed. **B** The SA-βGal staining and PAS staining of chondrocytes. Scale bars = 50 μm

To further identify the role of FBP1 in cartilage, we obtained primary chondrocytes from mice, used IL-1 to stimulate the chondrocytes to initiate OA, and then overexpressed FBP1 in granules, followed by measuring the expression of metabolic indicators of chondrocytes. The results showed that after stimulation with IL-1, with decreased expression of FBP1, levels of COL2, SOX9, and ACAN in chondrocytes were significantly decreased, while MMP13 and ColX increased (Fig. 2A). However, the overexpression group showed opposite results. We also used siFBP1 to knock-down the expression of FBP1 in primary chondrocytes, and then measured the same metabolic markers of chondrocytes, which showed that the results were the opposite of those from overexpression (Figure S1a). Together, these results showed that overexpression of FBP1 promoted anabolism and inhibited catabolism of cartilage cells. We also characterized the senescence of chondrocytes. Using PAS staining, we found that after overexpression of FBP1, the levels of glycogen and other polysaccharides of chondrocytes increased, suggesting that the inhibition of gluconeogenesis was alleviated. Furthermore, the results of β-glycoside staining showed that overexpression of FBP1 alleviated the senescence of chondrocytes (Fig. 2B). Taken together, these results indicated that FBP1 promoted the anabolism of chondrocytes, inhibited their catabolism, and delayed senescence.

### FBP1 suppresses the degeneration of cartilage and delays the progression of OA

To verify that FBP1 had a protective effect in cartilage, we constructed a cartilage external implant and induced degeneration using IL-1 stimulation, and then constructed a lentivirus that overexpressed FBP1, to treat the explant. Safranine O-Fast Green staining results showed that bulk degeneration of the lentivirus group was decreased. In addition, immunohistochemical studies showed that overexpression of FBP1 promoted cartilage synthesis, while inhibiting decomposition and aging, further indicating that FBP1 had a protective effect on cartilage (Figure S3a-e).

**Fig. 3:**
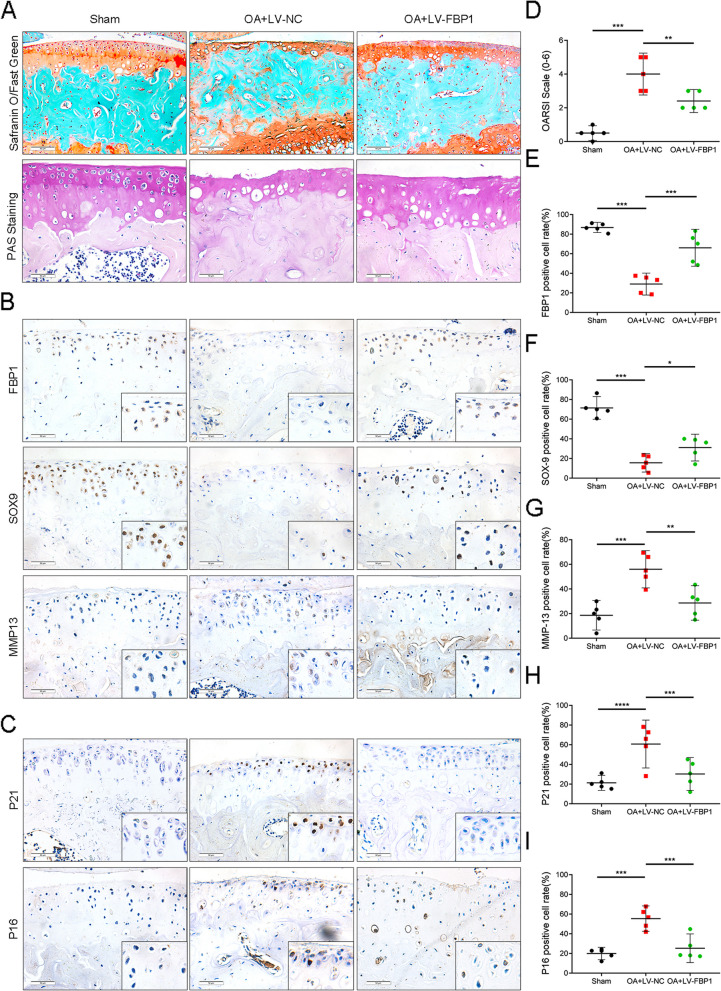
The overexpressing of FBP1 can suppress the degeneration of cartilage and delay the progression of OA. **A** Safranin O/fast green staining(first row), PAS staining (second row) images of knee cartilage from control group, destabilization of medial meniscus (DMM) group, and DMM with lentivirus group mice. Scale bars = 50 μm. **B,C** IHC staining of FBP1, SOX9, MMP13, P21, and P16 positive cells in articular cartilage of control group, DMM group, and DMM with lentivirus group mice. Scale bars = 50 μm. **D–I** Quantitative analysis of the OARSI scale and FBP1-positive, SOX9-positive, MMP13-positive, P21-positive, and P16-positive chondrocytes in mice. *n* = 5 per group. *p < 0.05, **p < 0.01, ***p < 0.001, NS = not significant. One-way analysis of variance (ANOVA) was performed

To confirm the role of FBP1, we conducted in vivo experiments. We established a DMM (destabilization of the medial meniscus) OA mice model (refer to materials and method) and altered the expression of FBP1 in cartilage by regular joint injections of lentivirus over a period of 8 weeks. Immunohistochemical staining of FBP1 confirmed that FBP1 of the lentivirus group was successfully overexpressed. The results of Safranine O-Fast Green staining showed that cartilage damage in mice injected with lentivirus was lower than that of the OA control group, indicating that FBP1 had a protective effect on joint cartilage. Moreover, we used PAS staining to show that the polysaccharide content of cartilage in the lentivirus group increased, which suggested that the inhibition of gluconeogenesis was alleviated (Fig. 3A). We also conducted immunohistochemical staining to detect changes in cartilage metabolic indicators and found that SOX9 increased, when compared with the OA control group, and that the MMP13, P21, and P16 indicator decreased, indicating that the level of anabolism in cartilage increased while catabolism decreased and senescence delayed (Fig. 3B,C). These results further verified that FBP1 had a protective effect on cartilage in OA.

### FBP1 suppresses OA progression through promotion of the CRB3

**Fig. 4:**
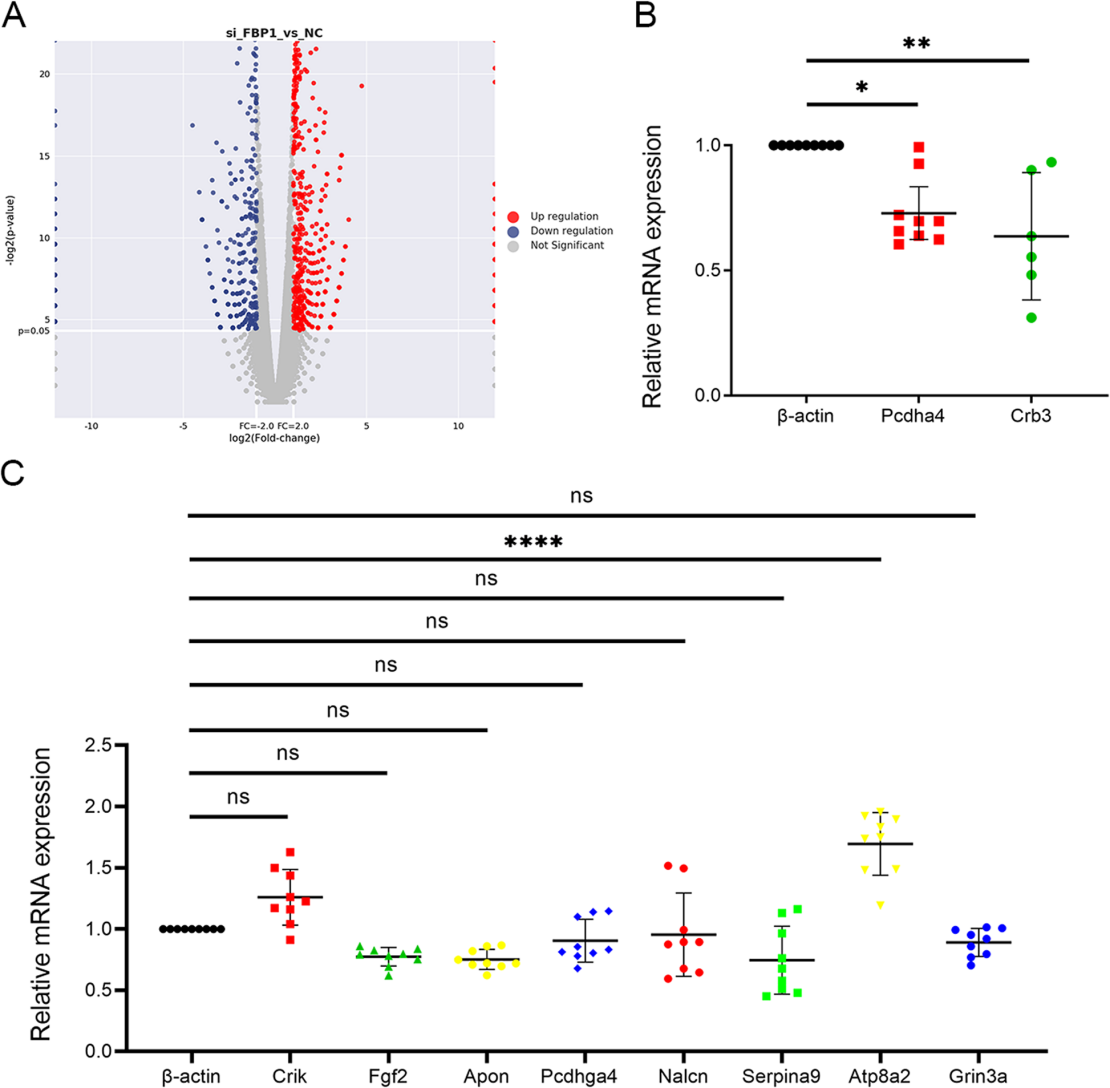
Genes that are downregulated with downregulation of FBP1 expression in chondrocytes. The volcano map (**A**) based on chondrocytes sequenced with siFBP1 treatment and 10 genes with the most significant decrease in expression were screened. **B, C** Quantitative PCR analysis of the 10 genes screened in mouse primary chondrocytes treated with or without siFBP1 for 48 h. *n* = 9 per time point. ***p < 0.001, NS = not significant. One-way analysis of variance (ANOVA) was performed

**Fig. 5:**
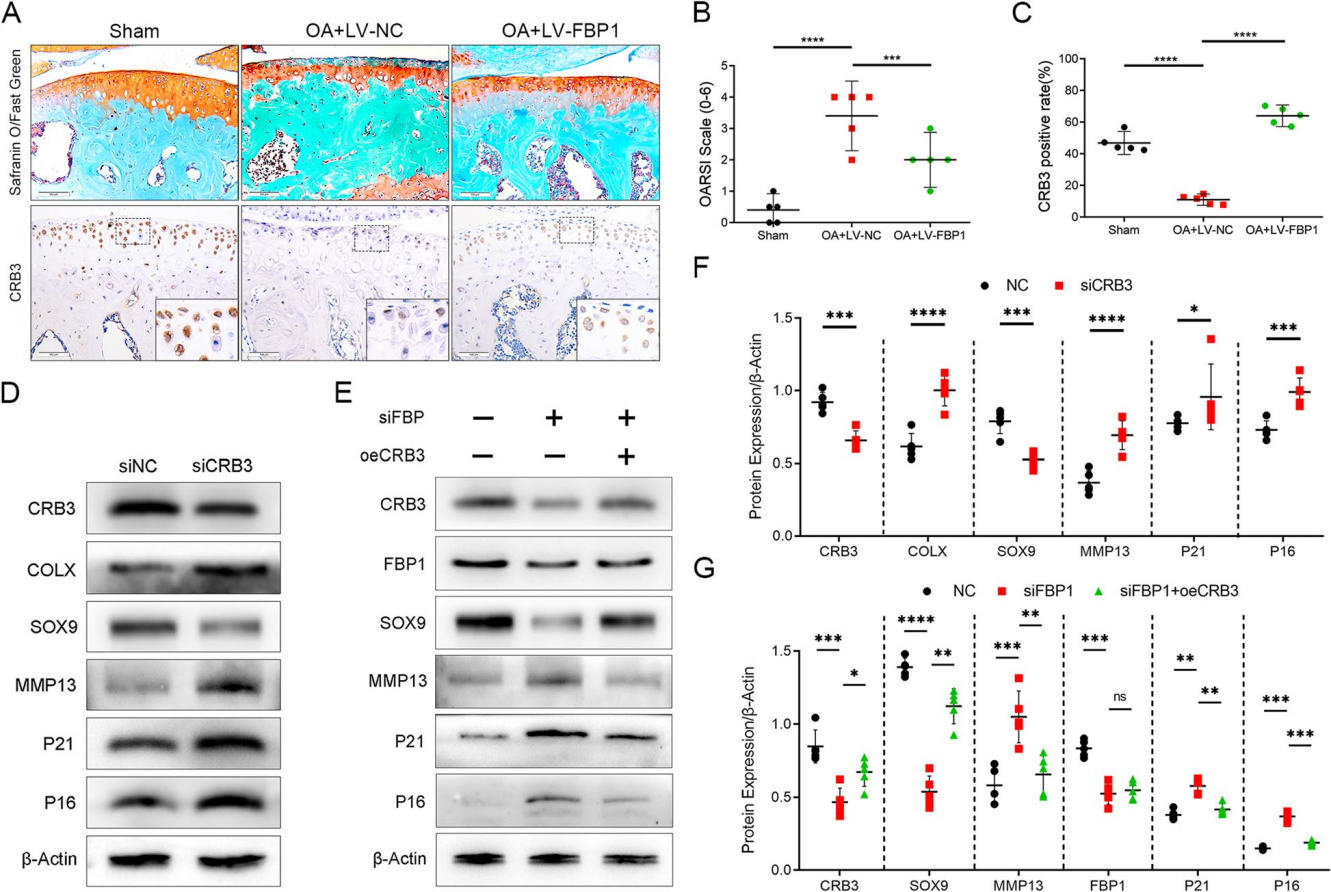
FBP1 suppresses OA progression through promotion of the CRB3. **A** The Safranin O and fast green staining of cartilage of mice and immunohistochemistry staining for CRB3. Scale bars = 100 μm. **B,C** The quantitative analysis of the OARSI scale and CRB3-positive chondrocytes in OA mice. *n* = 5 per group. **D,F** Western blot and quantification of COLX, CRB3, SOX9, MMP13, P21, and P16 in siCRB3-treated primary chondrocytes from mice. **E,G** Western blot and quantification of CRB3, SOX9, MMP13, P21, P16, and FBP1 in oeFBP1-treated primary chondrocytes from mice. *n* = 5 per group.*p < 0.05, **p < 0.01, ***p < 0.001, ****p < 0.0001, NS = not significant. One-way analysis of variance (ANOVA) was performed

**Fig. 6:**
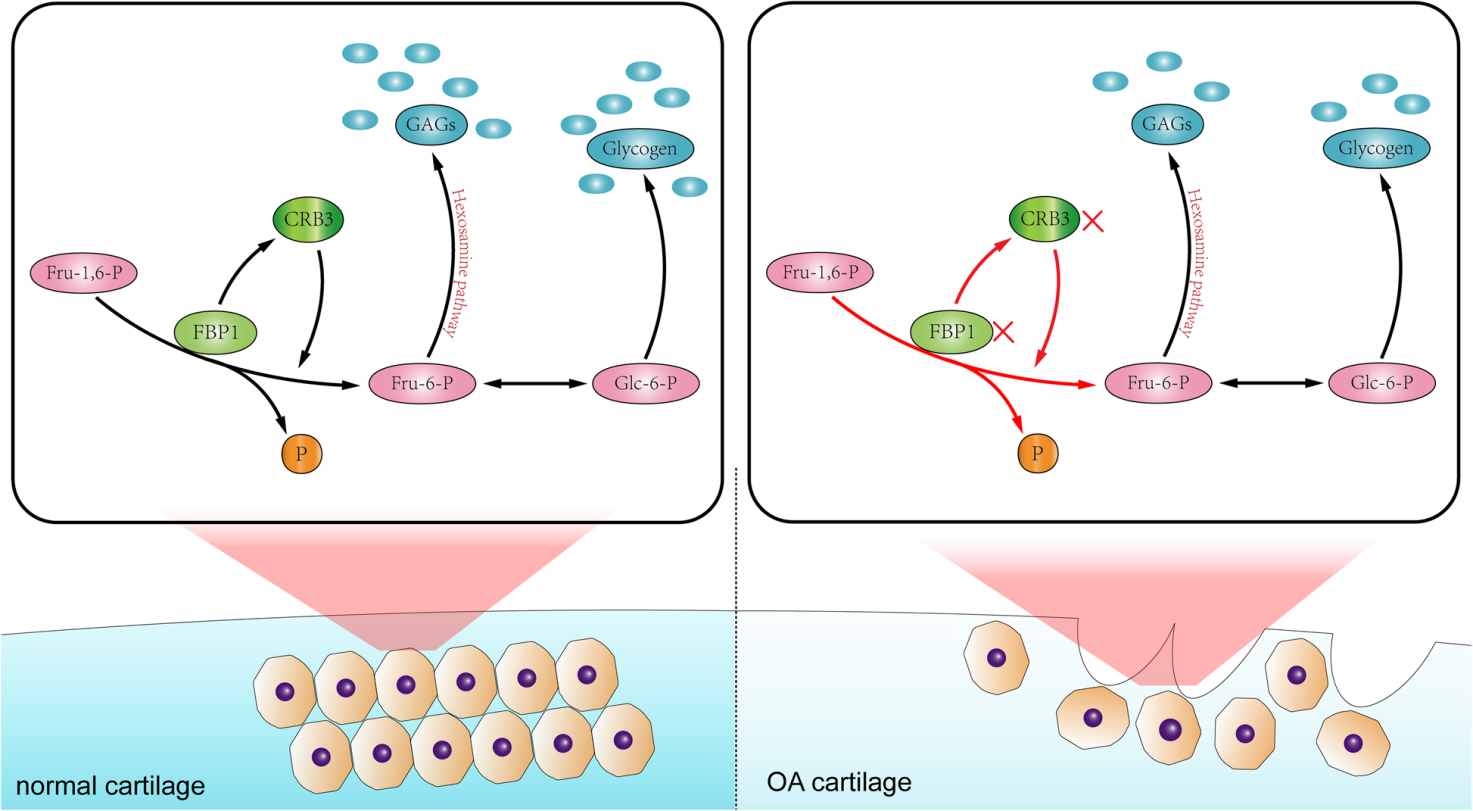
A model of gluconeogenesis disorder with decrease of gluconeogenesis key enzyme FBP1 and leading to impaired polysaccharide synthesis, cartilage destruction, and other effects during osteoarthritis. OA causes gluconeogenesis disorders, resulting in a decrease in FBP1, impaired the synthesis of glycogen, glycosaminoglycans, and other polysaccharide substances, and leads to a decrease in the expression of CRB3, aggravating the aging and degeneration of cartilage

We used siFBP1 to treat primary mice chondrocytes, and performed gene screening to identify the first 10 genes that were decreased (Fig. 4A,B). We verified their expressions using qPCR and found that Protein crumbs homolog 3 (CRB3) was the most expressed (Fig. 4C). To verify the expression of CRB3 in OA cartilage, we performed immunohistochemical staining of cartilage. The results showed that CRB3 was significantly expressed in OA cartilage (Figure S4a-c). Furthermore, in OA cartilage, which overexpressed FBP1 after lentivirus injection, CRB3 expression recovered (Fig. 5A–C). To further confirm the role of CRB3 in OA, we used siCRB3 to treat chondrocytes and extracted proteins for western blotting, which showed that after reduction of CRB3, the expression of SOX9 in chondrocytes declined, while the expressions of P16, P21, and MMP13 increased (Fig.  5D,F). We also performed the PAS staining and found that the glycogen and other polysaccharides of chondrocytes decreased (Figure S4d). These suggested that CRB3 has a protective effect on chondrocytes. To further verify the CRB3 relationship with FBP1, as well as its protective effect, we treated chondrocytes with siFBP1 and CRB3 overexpression plasmids and found that after the expressions of FBP1 and CRB3 were downregulated, SOX9 expression in chondrocytes decreased and expression of MMP13 increased. After overexpressing CRB3, the expression of SOX9 increased, while the expression of MMP13 decreased (Fig. 5E,G), suggesting that FBP1 inhibited the breakdown of chondrocytes by regulating CRB3, thereby alleviating cartilage degeneration and decreasing the progression of OA (Fig. 6).

## Discussion

In this study, we confirmed changes of gluconeogenesis during OA and found that during OA, glycogen, and other polysaccharide substances were significantly reduced and their synthesis was inhibited. We then confirmed expression changes of FBP1, a key enzyme of gluconeogenesis, and found that expression of FBP1 in OA tissues significantly decreased, further indicating that gluconeogenesis was inhibited during OA. In addition, we showed that FBP1 delayed cartilage degradation and chondrocyte aging, thereby alleviating OA. To better understand the mechanism of FBP1 in alleviating OA, we screened CRB3 and found that it alleviated OA. Overexpression of FBP1 through drugs or other means may therefore serve as a therapeutic approach to treat OA.

Carbohydrate metabolism plays an important role in the growth and development of cartilage, involving energy metabolism and anabolism of cartilage [30, 31]. Polysaccharide substances such as glycosaminoglycans are important components of cartilage, but require the consumption of large amounts of glucose and its derivatives, so the gluconeogenesis process that provides glucose is essential for the synthesis of polysaccharides [32]. In the present study, we showed that polysaccharides decreased in OA cartilage, indicating that gluconeogenesis was inhibited, which suggested that enzymes related to gluconeogenesis were inhibited. We speculated that inhibition of gluconeogenesis during OA inhibited the synthesis of glucosamine and other substances, thus affecting the repair of cartilage and causing cartilage degeneration [33, 34]. There is a study that found inhibition of glycolysis, which is the opposite way of gluconeogenesis, may be the new strategy for osteoarthritis [35]. However, the specific mechanism by which gluconeogenesis affects OA is unclear.

FBP1 is a rate-limiting enzyme for gluconeogenesis processes, which can regulate gluconeogenesis processes and is present in most cells, and it plays a tumor suppressor role in many cancer [36, 37]. Also, FBP1 can antagonize glycolysis that may be the new strategy for OA through its cytosolic catalytic activity [38, 39]. In the present study, we showed that expression of FBP1 was significantly reduced in OA cartilage of human and mouse. The result indicated that FBP1 may play a beneficial role in cartilage in OA. And after its overexpression, the levels of polysaccharide in cartilage increased, indicating that FBP1 promoted gluconeogenesis in cartilage. SOX9 is a protein necessary for cartilage formation, and MMP13 is a marker of cartilage catabolism [40, 41]. Therefore, to identify the effect of FBP1 on cartilage, we overexpressed FBP1 in OA cartilage and found that the destruction of articular cartilage was alleviated, while treatment with FBP1 increased the expression of SOX9 and downregulated the expression of MMP13. These results showed that FBP1 alleviated the destruction of cartilage during OA by promoting anabolism and inhibiting catabolism of cartilage. We also found that FBP1 lowered P16 and P21 levels of cartilage, which suggested that cartilage aging was reduced.

CRB3 is a protein expressed on the cell membrane that is involved in the polarity formation of cells and is also involved in physiological processes such as contact inhibition [42, 43]. CRB3 is closely related to the occurrence of some tumors such as colorectal carcinoma, breast cancer and oral squamous cell carcinomas and CRB3 affects the occurrence of these diseases by regulating the Hippo pathway [44–46]. In the present study, we found that there were many genes that were downregulated with FBP1 expression, among which CRB3 was the most obvious. We then confirmed that CRB3 was downregulated in OA, but also showed that knocking-down CRB3 promoted chondrocyte degeneration and aging and decreased the glycogen and other polysaccharides, while overexpression of CRB3 delayed the degeneration of chondrocytes caused by downregulation of FBP1; but it did not seem to reverse the decrease of FBP1 itself. These results suggested that FBP1 alleviated the degeneration and aging of chondrocytes during OA by regulating CRB3 (Fig. 6). Regarding its mechanism, studies have reported that CRB3 inhibited β-catenin, and the activation of this pathway led to apoptosis and aging of chondrocytes, which may be the reason why it alleviated OA cartilage degeneration [47]. β-catenin can also affect the carbohydrate metabolism and glucose transport [48, 49]. CRB3 could affect Hippo pathway and some study showed that Hippo pathway contributed to osteoarthritis and that it could regulate the glycometabolism in mice [50, 51]. CRB3 may therefore delay cartilage degeneration and affect carbohydrate metabolism by regulating β-catenin pathway and Hippo pathway, to slow the progression of OA.

Although this study described the effects of glucose metabolism and FBP1 on OA, there were still shortcomings. In the study of FBP1, there was no convincing conclusion as to whether FBP1 affected OA through glucose metabolism, or whether glucose metabolism changes was only a phenotype of OA. In addition, although we showed that FBP1 affected OA downstream molecules, the pathway affected was not identified. Furthermore, regarding the role of CRB3, the effect of CRB3 on OA has not been fully verified in vivo, and the relationship between CRB3 and gluconeogenesis processes has not been elucidated.

## Conclusion

In this study, we confirmed changes of gluconeogenesis during OA and found that during OA, glycogen and other polysaccharide substances were significantly reduced and their synthesis was inhibited. We then confirmed expression changes of FBP1, a key enzyme of gluconeogenesis, and found that expression of FBP1 in OA tissues significantly decreased, further indicating that gluconeogenesis was inhibited during OA. In addition, we showed that FBP1 delayed cartilage degradation and chondrocyte aging, thereby alleviating OA. To better understand the mechanism of FBP1 in alleviating OA, we screened CRB3 and found that it alleviated OA. Overexpression of FBP1 through drugs or other means may therefore serve as a therapeutic approach to treat OA.

## Materials and methods

### Human cartilage tissue

Healthy human articular cartilage tissue was obtained from the victims of traffic accidents, with no history of arthritic diseases (*n* = 5, age 30.6 ± 9.22 years, four males and one females). OA cartilage tissue was obtained from OA patients (*n* = 5, age: 64 ± 5.75 years, two males, three females) undergoing total knee joint replacements. Other joint diseases were excluded from the study. All samples were from the Department of Orthopedics, the Third Affiliated Hospital of Southern Medical University. All recruited patients contributed their informed consent and were identified and approved by the Ethics Committee of the Third Affiliated Hospital of Southern Medical University.

### Animals and the mouse model

All animal experiments were approved by the Animal Care and Use Committee of Southern Medical University. Six-week-old male C57BL/6 mice were purchased from the Experimental Animal Center of Southern Medical University, Guangzhou, China.

In the instability of medial meniscus OA model (DMM, Kamekura et al., 2005) [52], mice were anesthetized by intraperitoneal injection of 5% chloral hydrate, and the skin was cut along the medial collateral ligament. The joint capsule was opened to expose the femoral condyle. Then, the connection between the medial meniscus and the tibial plateau was cut open to release the medial meniscus. The joint capsule and skin were sutured after surgery.

### Animal treatment and specimen preparation

After surgery, 9 × 10^8^ TU/mL (total volume of approximately 5 µL) was injected into the joint once a week using OeFBP1 lentivirus. The right leg was then collected from 4 to 8 weeks after surgery (*n* = 5 in each group). The knee joints of mice in different experimental groups were fixed in 4% paraformaldehyde for 48 h and decalcified for 21 days. The specimens were then embedded in paraffin, and 4 μm serial sections were cut from the sagittal portion through the inner side of the knee.

### Cell and cartilage explants

The prechondral cell line, ATDC5 (Tsukuba, Japan), was cultured in DMEM/F12 (Gibco, Grand Island, NY, USA), containing 10% fetal bovine serum (FBS; Gibco), 100 U/mL penicillin, and 100 mg/mL streptomycin sulfate (Gibco) at 37 °C, in an incubator containing 5% CO_2_. We dissected the rib cartilages of newborn mice (24 − 72 h of age) under a stereoscopic optical microscope to obtain primary chondrocytes. After trypsin digestion for 30 min, primary chondrocytes were isolated and purified, then digested at 37 °C for 4 − 6 h using 0.1% type II collagenase (Sigma-Aldrich, St. Louis, MO, USA) containing 10% FBS, 100 U/mL penicillin, and 100 mg/mL streptomycin sulfate. The primary chondrocytes were then resuspended and inoculated in 24-well plates and cultured in DMEM/F 12 containing 10% FBS, 100 U/mL penicillin, and 100 mg/mL streptomycin sulfate at 37 °C and 5% CO_2_. The femoral head was separated from 8-week-old mice. The explants cultured in DMEM/F12 were treated with 10 mM phosphate-buffered saline (PBS) for 1 h, followed by 50 ng/mL recombinant mouse or human interleukin (IL-1β) in PBS/0.1% bovine serum albumin (BSA) or PBS/0.1% BSA alone. The explants were then harvested and the culture medium was collected after 3 days of culture.

### Real‐time polymerase chain reaction

Total RNA was isolated from and cartilage of the human tibial plateau, then ground using TRIzol reagent. For mRNA quantification, 1 mg of total RNA was purified with gDNA remover and transcribed using 5 × HiScript II qRT SuperMix II (Vazyme Biotech, Nanjing, China). Each PCR reaction consisted of 10 μL 2 × ChamQ SYBR qPCR main mixture, 10 μM forward and reverse primers, and 500 ng cDNA. For miRNA quantification, 1 mg of total RNA was purified using a gDNA wipe mixture, followed by a Hiscript II enzyme mixture of 10 × RT mixture and specific stem ring primers for reverse transcription. Template DNA was combined with 2 × Mix miRNA Universal SYBR qPCR Master Mix, specific primers, and mQ primer R. All reactions were in triplicate. Primer sequences were of mice and are listed below:

Fbp1.

forward 5′- TGCTGAAGTCGTCCTACGCTAC-3′,

reverse 5′- TTCCGATGGACACAAGGCAGTC-3′;

Sox9.

forward 5′- CACACGTCAAGCGACCCATGAA-3′,

reverse 5′- TCTTCTCGCTCTCGTTCAGCAG-3′;

Col2.

forward 5′- GCTGGTGAAGAAGGCAAACGAG-3′,

reverse 5′- CCATCTTGACCTGGGAATCCAC-3′;

Acan.

forward 5′- CAGGCTATGAGCAGTGTGATGC-3′,

reverse 5′- GCTGCTGTCTTTGTCACCCACA-3′;

Mmp13.

forward 5′- GATGACCTGTCTGAGGAAGACC-3′,

reverse 5′- GCATTTCTCGGAGCCTGTCAAC-3′;

ColX.

forward 5′- GTACCAAACGCCCACAGGCATA-3′,

reverse 5′- GGACCAGGAATGCCTTGTTCTC-3′;

Crik.

forward 5′- TGTCTGGCTGTCTGGAACTC-3′,

reverse 5′- GAAGGACAATGGGCATCATGG-3′;

Fgf2.

forward 5′- AGCGGCTCTACTGCAAGAAC-3′,

reverse 5′- GTTGGCACACACTCCCTTGA-3′;

Apon.

forward 5′- AGGCTGATGAGTAGCCCAGA-3′,

reverse 5′- CGTCTAGCTACACACCGTGG-3′;

Pcdha4.

forward 5′- CTGATTCAAGGGACAGAGAGGA-3′,

reverse 5′- CTGGACCAGCCCGTAGAATG-3′;

Pcdhga4.

forward 5′- CCAGCGCTTGCTTCTTTCTT-3′,

reverse 5′- GCCTCCTCAGGGATGGAGTA-3′;

Nalcn.

forward 5′- CAACAGCAAAAGGCAAGCGA-3′,

reverse 5′- ACCACAGTCTGTAACCGCAG-3′;

Serpina9.

forward 5′- TGAGGTGAGCACTCAGACAC-3′,

reverse 5′- TGTCCCTAACCCTGAACCGT-3′;

Atp8a2.

forward 5′- TTCTGCGGGCTACAAGAAGG-3′,

reverse 5′- TACTGATCCGGTTGTCGCAG-3′;

Crb3.

forward 5′- GGGTGACTAAACTTTCCGGGT-3′,

reverse 5′- GACTTCGCTCAGGTTCCCAA-3′;

Grin3a.

forward 5′- CCGCAACTCCCTCACCTATC-3′,

reverse 5′- GAATGGCTTGGAGTGTGGGA-3′;

### Western blotting

Pyrolysis buffer was comprised of 10% glycerol, 2% sodium dodecyl sulfate (SDS), 10 mM dithiothreitol, 10 mM Tris–HCl (pH 6.8), 1 mM phenymethylsulfonlyfloride, and 10% ethanethiol. The proteins were incubated in pyrolysis buffer at 98 °C for 10 min. The sample was resolved using SDS-PAGE for 90 min. The sample was then transferred to a nitrocellulose membrane for 1 h and incubated with primary antibody (in 5% BSA, 0.2% NaN_3_) at 4 °C overnight. The primary antibodies are mouse anti-β-Actin (1:10,000), rabbit anti-SOX9 (1:2000), rabbit anti-MMP13 (1:2000), rabbit anti-COLX (1:2000), mouse anti-P21 (1:1000), mouse anti-P16 (1:1000), rabbit anti-FBP1 (1:200), and mouse anti-CRB3 (1:1000). Samples were then incubated with secondary antibodies (mouse or rabbit, 1:4000) at 37 °C for 1 h.

### Histology and immunohistochemical/IF staining

Morphological analysis was performed on tissue sections using Safranine O-Fast Green staining. Immunohistochemical (IHC) and immunofluorescence (IF) staining were performed on 0.004-mm-thick tissue sections. The glass slide was dewaxed and rehydrated and washed three times in PBS for 5 min each time. The slide was then soaked in citric acid buffer and heated in a microwave for 2 min to recover the antigen. After washing three times in PBS, the slide was quenched at room temperature in 3% hydrogen peroxide for 10 min and washed three more times with PBS. Then, the slides were blocked with 10% BSA for 1 h at room temperature (IHC staining). Slides were then incubated with primary antibodies at 4 °C overnight. A secondary antibody for IHC or a fluorescent secondary antibody for IF was then used at room temperature for 1 h. Then, IHC slides were stained with diaminobenzidine and hematoxylin, dehydrated, and fixed. The IF slide was then treated with 4,6-diamino-2-phenylindole staining solution and fixed with a cover glass. Antibodies used for IHC/IF staining were as follows: rabbit anti-FBP1, rabbit anti-SOX9, rabbit anti-MMP13, rabbit anti- P21, rabbit anti-P16, rabbit anti-Aggrecan, rabbit anti-ColX, mouse anti-CRB3, species-matched horseradish peroxidase-conjugated secondary antibodies, and species-matched Alexa-488-or 594-labeled secondary antibody (rabbit 1:200).

### Statistical analysis

Data are expressed as the mean ± standard deviation. Unpaired Student’s *t* test was used for experiments comparing two groups of data. One-way analysis of variance was performed for data involving multiple groups, followed by Tukey’s post hoc test. A value of *p* < 0.05 was considered statistically significant.

### Supplementary Information

**Additional file 1:** **Figure S1.** FBP1 restores chondrocytes homeostasis and delay its aging in OA. (A) Quantitative PCR analysis of FBP1, SOX9, COL2A1, ACAN, MMP13 and ColX in mouse primary chondrocytes treated with or without granules that decrease the expression of FBP1. *n* = 5 per time point. *p < 0.05, **p < 0.01, ***p < 0.001, ****p < 0.0001,NS = not significant. One-way analysis of variance (ANOVA) was performed.

** Additional file 2:** **Figure S2.** The efficacy of lentivirus-FBP1 in explant and cartilage of mice. (A) Representative images of GFP of lentivirus-FBP1 in explant and cartilage of mice. Scale bars = 50 μm.

** Additional file 3:** **Figure S3.** The overexpressing of FBP1 can suppress the degeneration of cartilage and delay the progression of OA. (A) Representative images of safranin O/fast green staining(first row) and IHC staining of FBP1,SOX9,MMP13 positive cells in articular cartilage of IL-1β treated, IL-1β with lentivirus treated, and Control explants. Scale bars = 50 μm. (B,C,D,E) Quantitative analysis of the OARSI scale and FBP1-positive,SOX9-positive,MMP13-positive chondrocytes in explants. n = 5 per group.*p < 0.05, **p < 0.01, ***p < 0.001, ****p < 0.0001, NS = not significant. One-way analysis of variance (ANOVA) was performed.

** Additional file 4:** **Figure S4.** CRB3 decreased in articular cartilage in OA and down-regulated CRB3 could decrease the glycogen and other polysaccharides of chondrocytes. (A) Representative images of safranin O/fast green staining(first row) and IHC staining of CRB3 in human articular cartilage. Scale bars = 100 μm. (B,C) Quantitative analysis of the OARSI scale and CRB3-positive chondrocytes in human cartilage. *n* = 5 per group. *p < 0.05, **p < 0.01, ***p < 0.001. One-way analysis of variance (ANOVA) was performed. (D)PAS staining of chondrocytes treated by siCRB3 or siNC. Scale bars = 25 μm.

** Additional file 5.** 

## Data Availability

The datasets used and/or analyzed during the current study are available from the corresponding author on reasonable request.

## Abbreviations

OA: Osteoarthritis
FBP1: Fructose-bisphosphatase1
ECM: Extracellular matrix
MMP13: Matrix metalloproteinase
PAS staining: Periodic acid Schiff staining
DMM: Destabilization of the medial meniscus
CRB3: Protein crumbs homolog 3
