# Correction: Fructose‑bisphosphatase1 (FBP1) alleviates experimental osteoarthritis by regulating Protein crumbs homolog 3 (CRB3)

**DOI:** 10.1186/s13075-023-03247-9

**Published:** 2023-12-28

**Authors:** Zhuolun Wang, Xinjie Wang, Liangliang Liu, Xiongtian Guo, Haiyan Zhang, Jianbing Yin, Rengui Lin, Yan Shao, Daozhang Cai

**Affiliations:** 1 Department of Orthopedics, Orthopedic Hospital of Guangdong Province, Academy of Orthopedics·Guangdong Province, Guangdong Provincial Key Laboratory of Bone and Joint Degeneration Diseases, The Third Affiliated Hospital of Southern Medical University, Guangzhou, 510630 Guangdong China; 2 Department of Joint Surgery, Center for Orthopedic Surgery, Orthopedic Hospital of Guangdong Province, The Third School of Clinical Medicine, Southern Medical University, The Third Affiliated Hospital of Southern Medical University, Guangzhou, Guangdong China

**Correction: Arthritis Res Ther 25, 235 (2023)**

**https://doi.org/10.1186/s13075-023-03221-5**

Following publication of the original article [1], the authors reported that the following Equal Contribution note was missing in the article “Zhuolun Wang, Xinjie Wang, Liangliang Liu and Xiongtian Guo contributed equally to this manuscript”.

The original article [1] has been updated.
