# Supplementary figures and images for: Fructose-bisphosphatase1 (FBP1) alleviates experimental osteoarthritis by regulating Protein crumbs homolog 3 (CRB3)
Source: Arthritis Res Ther. 2023 Dec 4;25:235. doi: 10.1186/s13075-023-03221-5 (PMC10694907; doi:10.1186/s13075-023-03221-5)

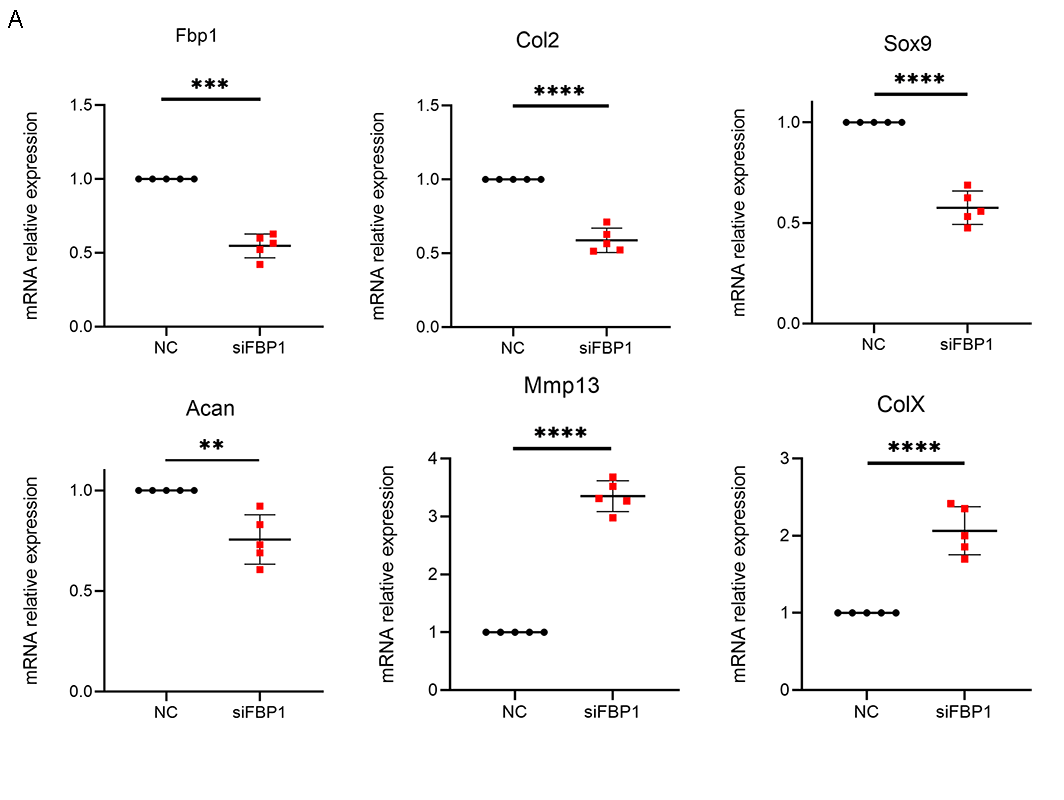

Supplement: Supplementary file 1 — Additional file 1: Figure S1. FBP1 restores chondrocytes homeostasis and delay its aging in OA. (A) Quantitative PCR analysis of FBP1, SOX9, COL2A1, ACAN, MMP13 and ColX in mouse primary chondrocytes treated with or without granules that decrease the expression of FBP1. n = 5 per time point. *p < 0.05, **p < 0.01, ***p < 0.001, ****p < 0.0001,NS = not significant. One-way analysis of variance (ANOVA) was performed. [file 13075_2023_3221_MOESM1_ESM.tif]

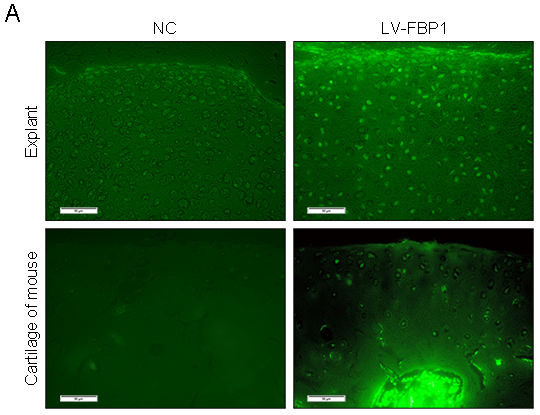

Supplement: Supplementary file 2 — Additional file 2: Figure S2. The efficacy of lentivirus-FBP1 in explant and cartilage of mice. (A) Representative images of GFP of lentivirus-FBP1 in explant and cartilage of mice. Scale bars = 50 μm. [file 13075_2023_3221_MOESM2_ESM.tif]

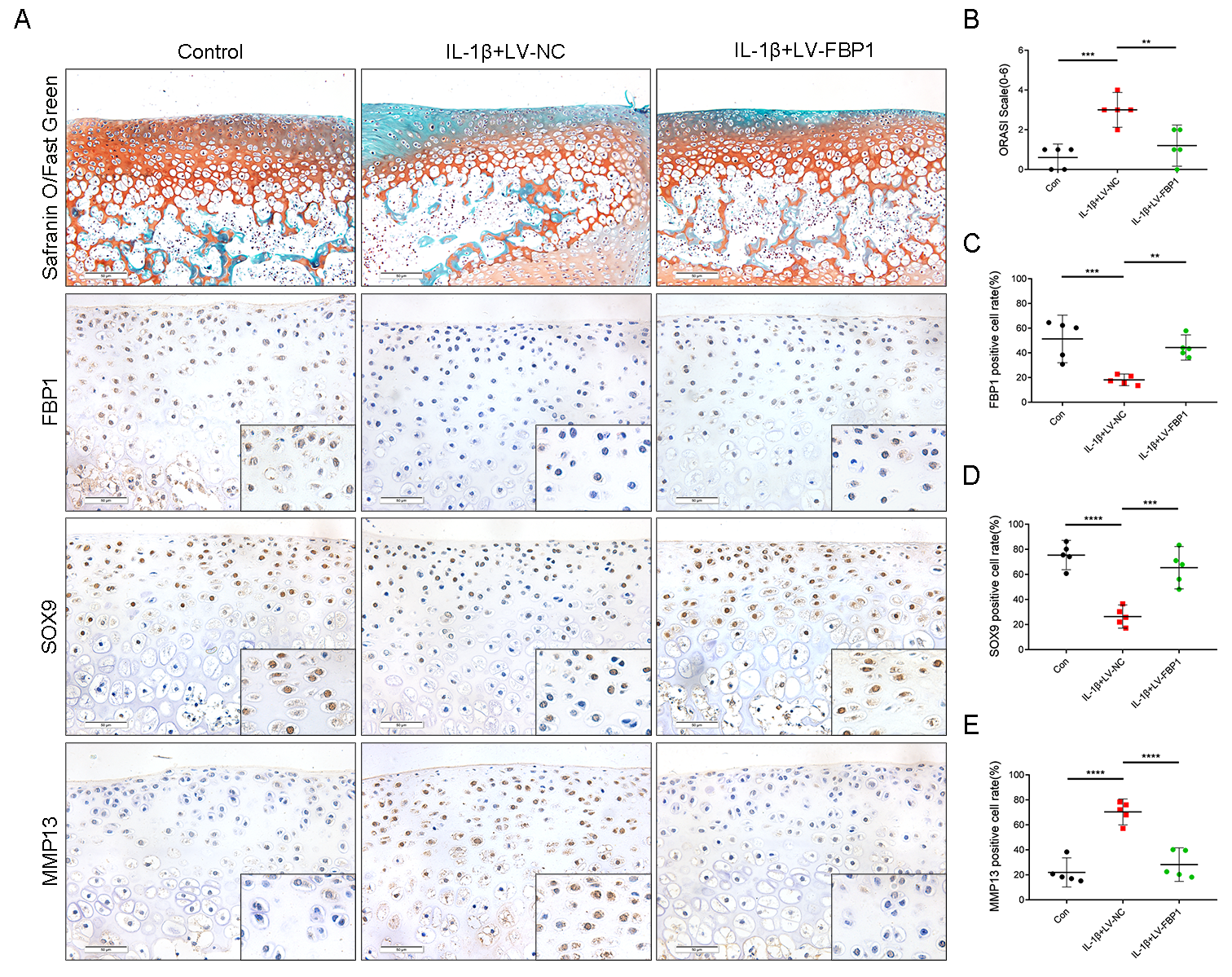

Supplement: Supplementary file 3 — Additional file 3: Figure S3. The overexpressing of FBP1 can suppress the degeneration of cartilage and delay the progression of OA. (A) Representative images of safranin O/fast green staining(first row) and IHC staining of FBP1,SOX9,MMP13 positive cells in articular cartilage of IL-1β treated, IL-1β with lentivirus treated, and Control explants. Scale bars = 50 μm. (B,C,D,E) Quantitative analysis of the OARSI scale and FBP1-positive,SOX9-positive,MMP13-positive chondrocytes in explants. n = 5 per group.*p < 0.05, **p < 0.01, ***p < 0.001, ****p < 0.0001, NS = not significant. One-way analysis of variance (ANOVA) was performed. [file 13075_2023_3221_MOESM3_ESM.tif]

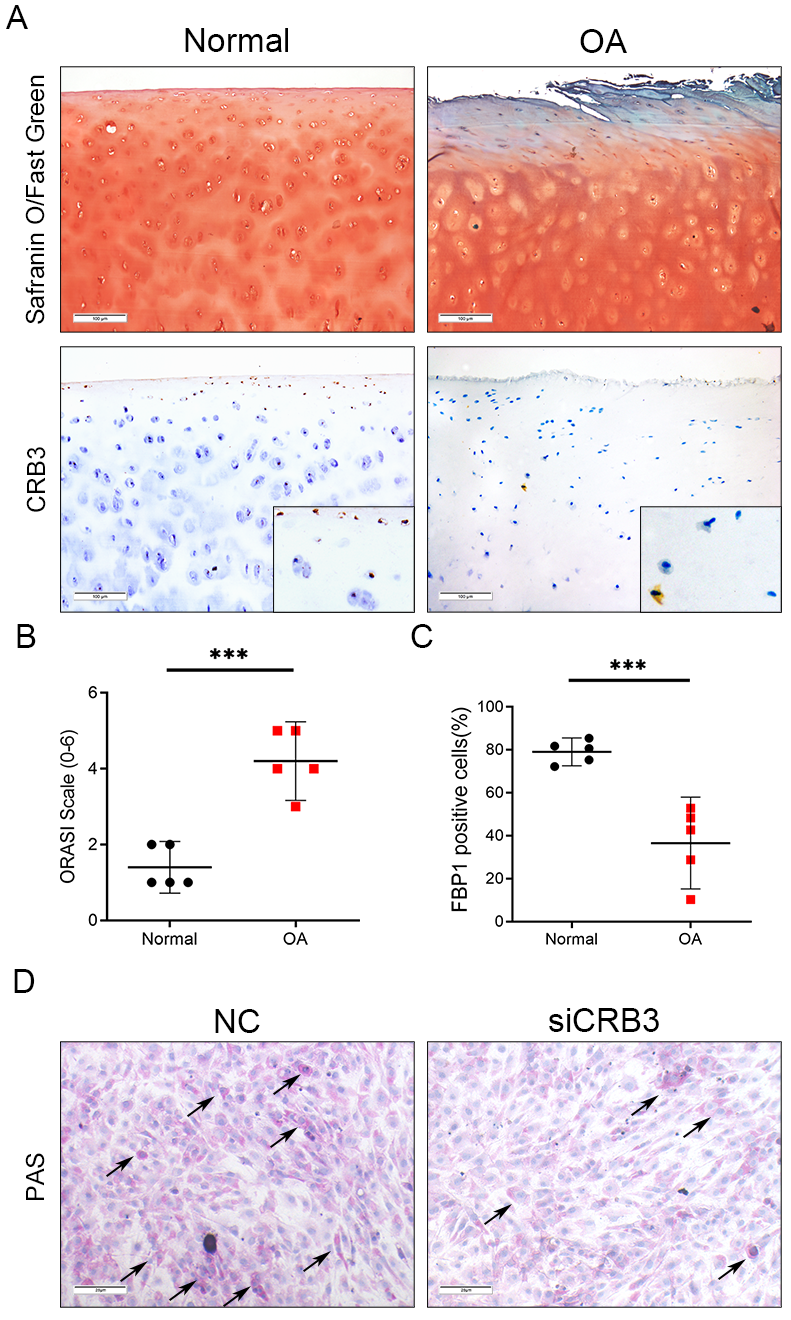

Supplement: Supplementary file 4 — Additional file 4: Figure S4. CRB3 decreased in articular cartilage in OA and down-regulated CRB3 could decrease the glycogen and other polysaccharides of chondrocytes. (A) Representative images of safranin O/fast green staining(first row) and IHC staining of CRB3 in human articular cartilage. Scale bars = 100 μm. (B,C) Quantitative analysis of the OARSI scale and CRB3-positive chondrocytes in human cartilage. n = 5 per group. *p < 0.05, **p < 0.01, ***p < 0.001. One-way analysis of variance (ANOVA) was performed. (D)PAS staining of chondrocytes treated by siCRB3 or siNC. Scale bars = 25 μm. [file 13075_2023_3221_MOESM4_ESM.tif]

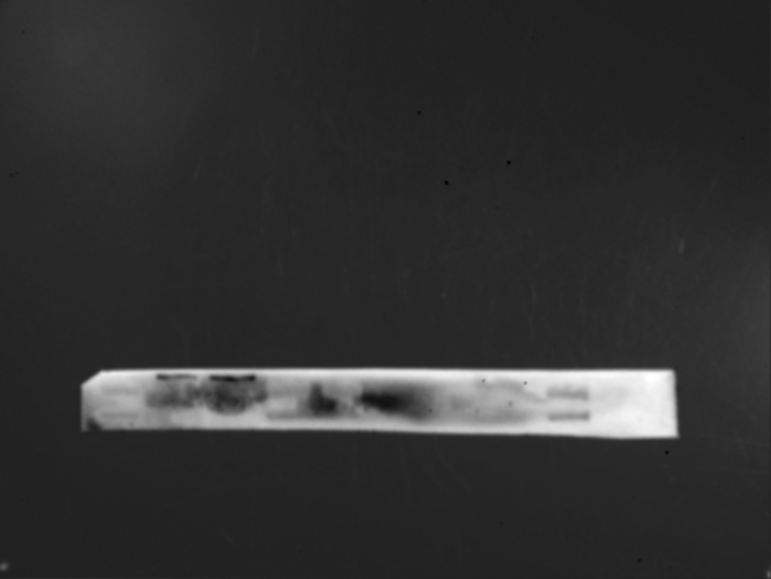

Supplement: Supplementary file 5 — Additional file 5. [file 13075_2023_3221_MOESM5_ESM.zip › Supplement4/Figure5D/COLX.tif]

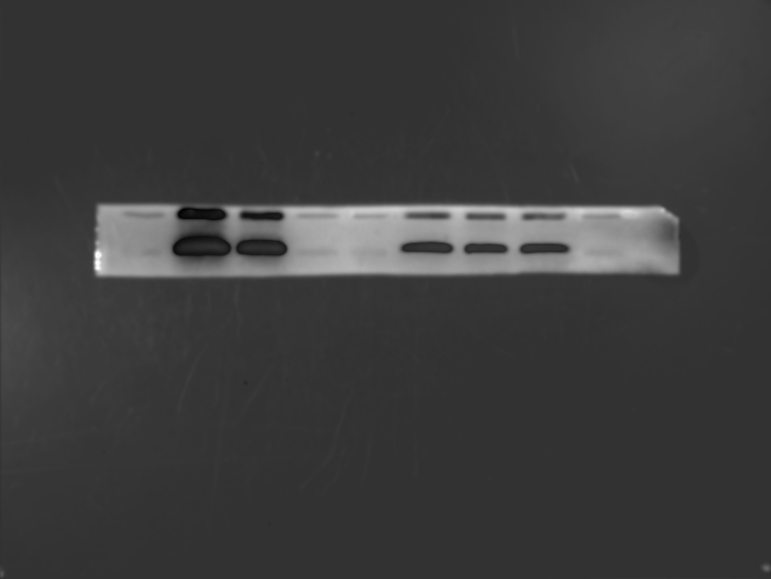

Supplement: Supplementary file 5 — Additional file 5. [file 13075_2023_3221_MOESM5_ESM.zip › Supplement4/Figure5D/CRB3.tif]

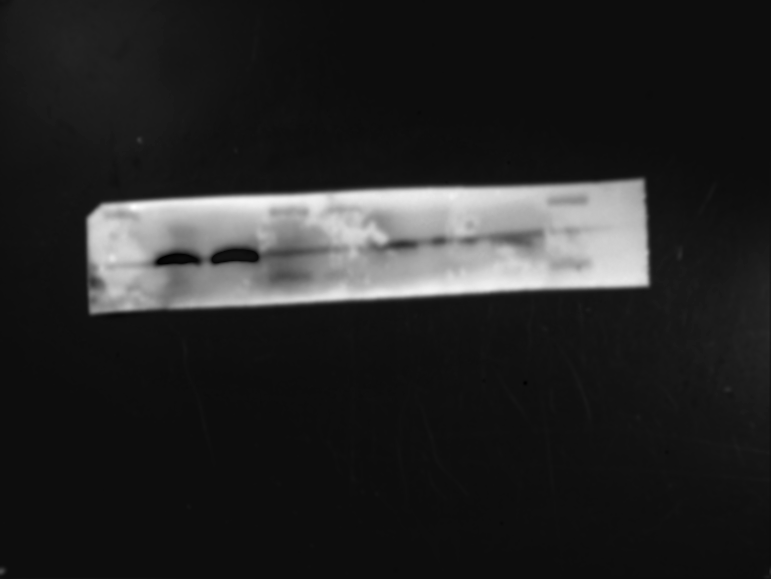

Supplement: Supplementary file 5 — Additional file 5. [file 13075_2023_3221_MOESM5_ESM.zip › Supplement4/Figure5D/P16.tif]

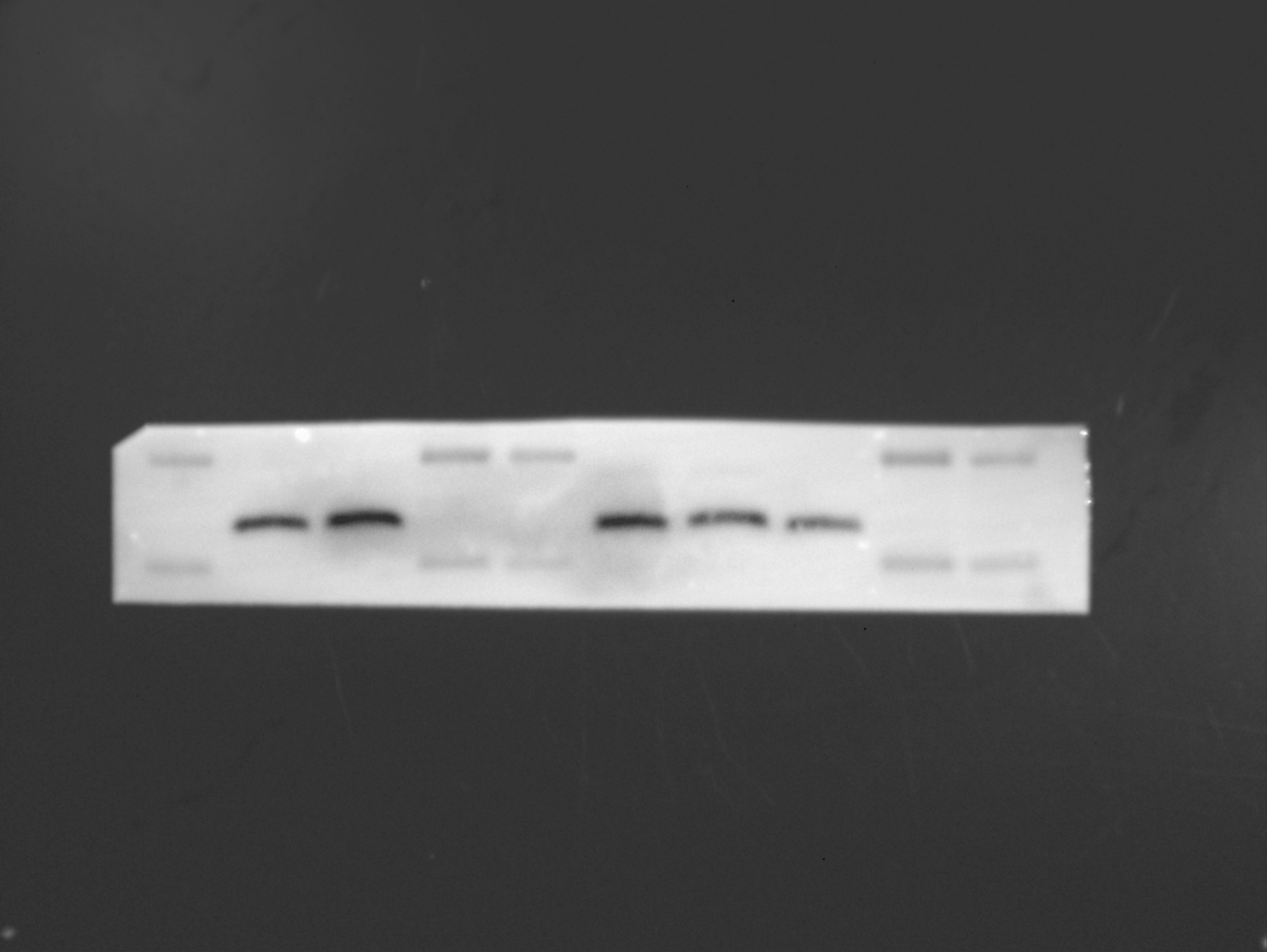

Supplement: Supplementary file 5 — Additional file 5. [file 13075_2023_3221_MOESM5_ESM.zip › Supplement4/Figure5D/P21.tif]

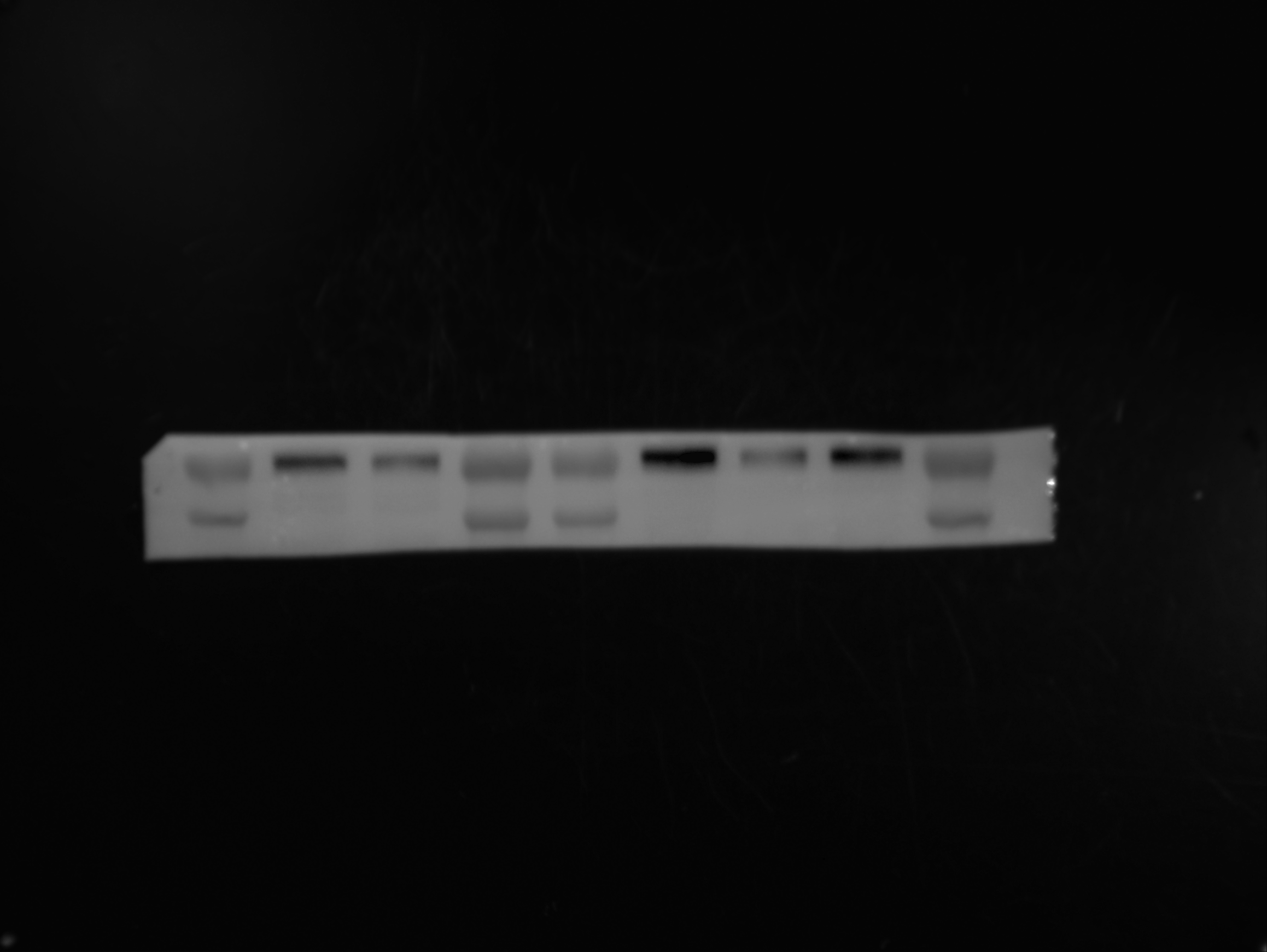

Supplement: Supplementary file 5 — Additional file 5. [file 13075_2023_3221_MOESM5_ESM.zip › Supplement4/Figure5D/SOX9.tif]

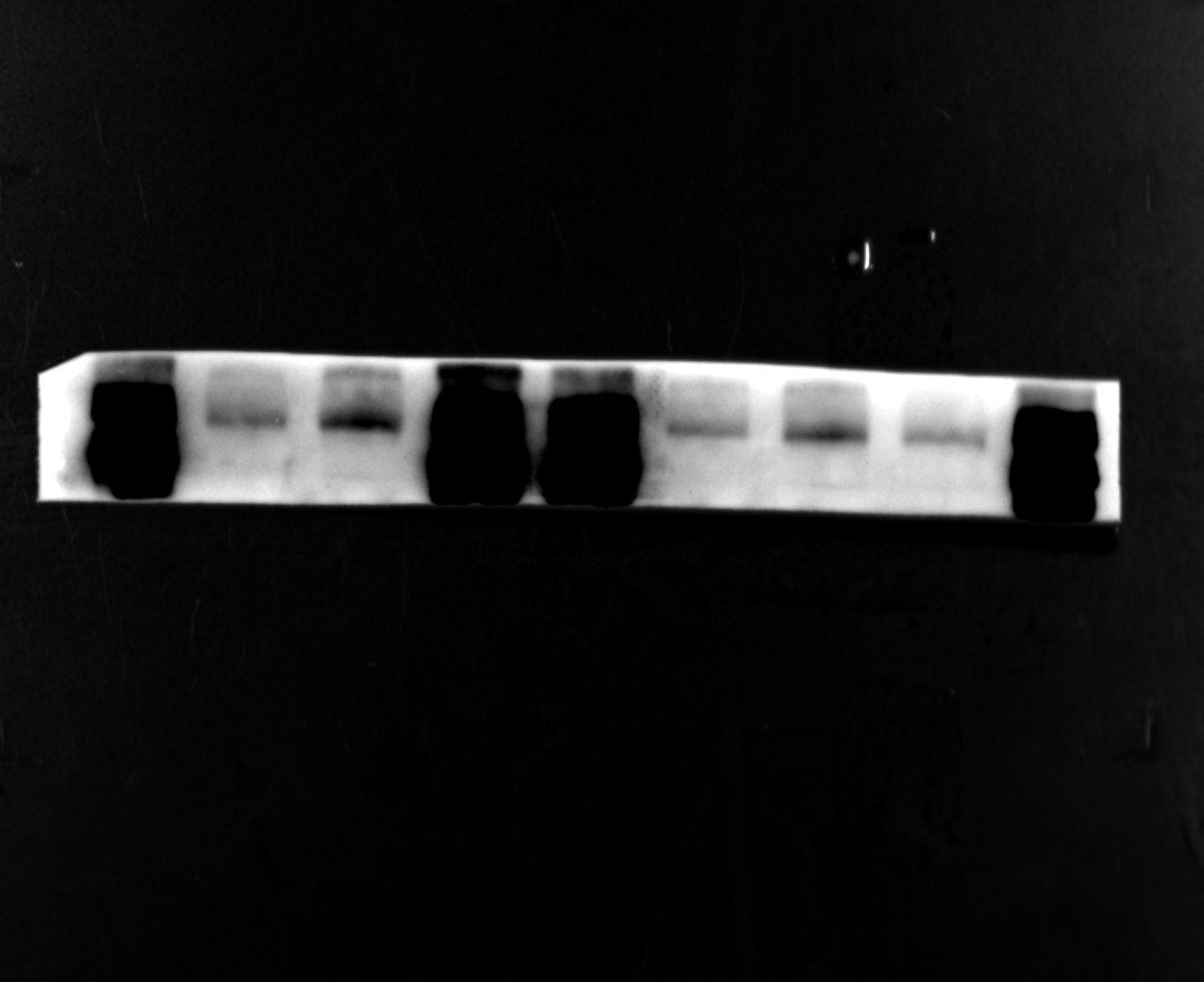

Supplement: Supplementary file 5 — Additional file 5. [file 13075_2023_3221_MOESM5_ESM.zip › Supplement4/Figure5D/mmp13.Tif]

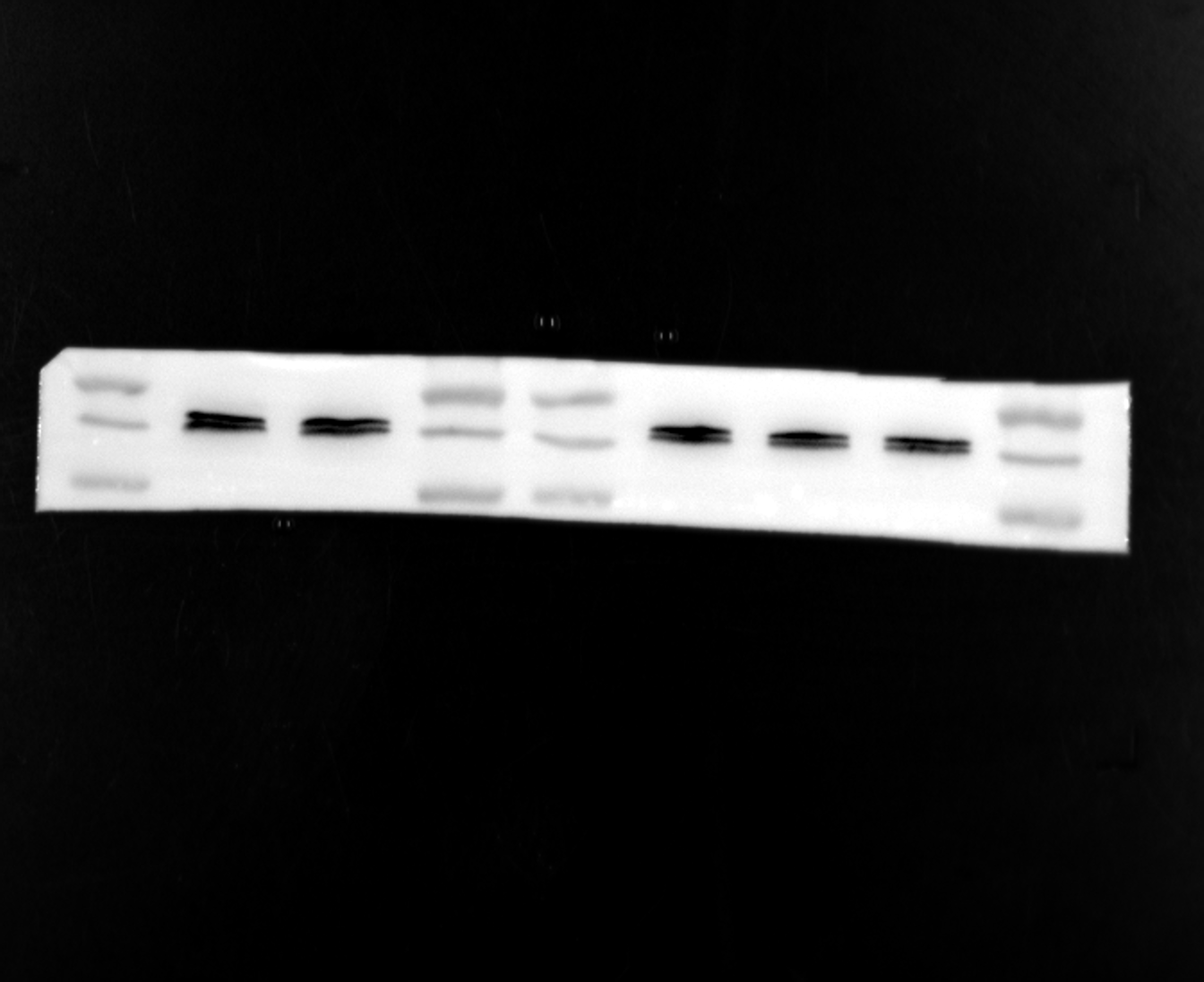

Supplement: Supplementary file 5 — Additional file 5. [file 13075_2023_3221_MOESM5_ESM.zip › Supplement4/Figure5D/β-actin(2).Tif]

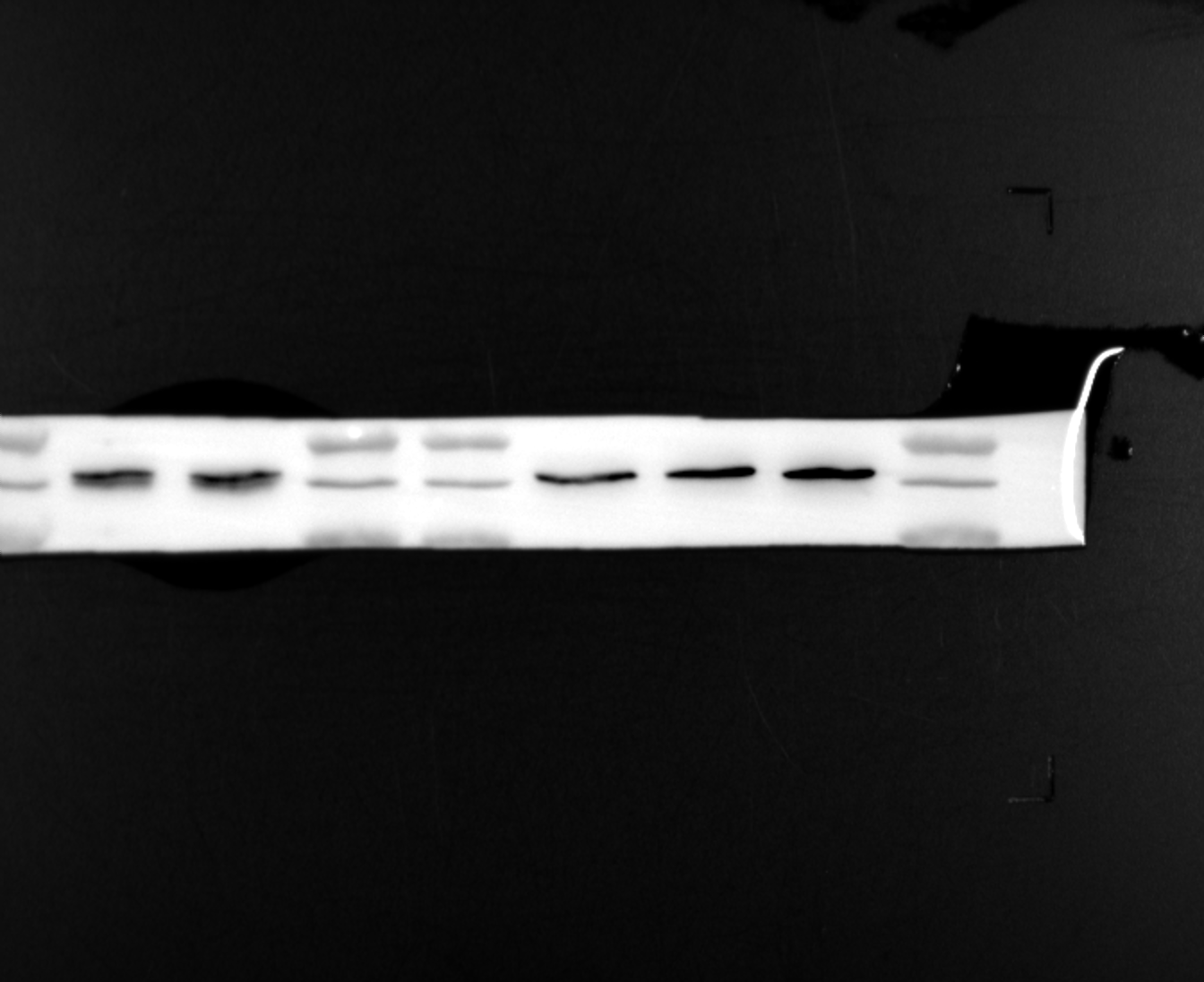

Supplement: Supplementary file 5 — Additional file 5. [file 13075_2023_3221_MOESM5_ESM.zip › Supplement4/Figure5D/β-actin.Tif]

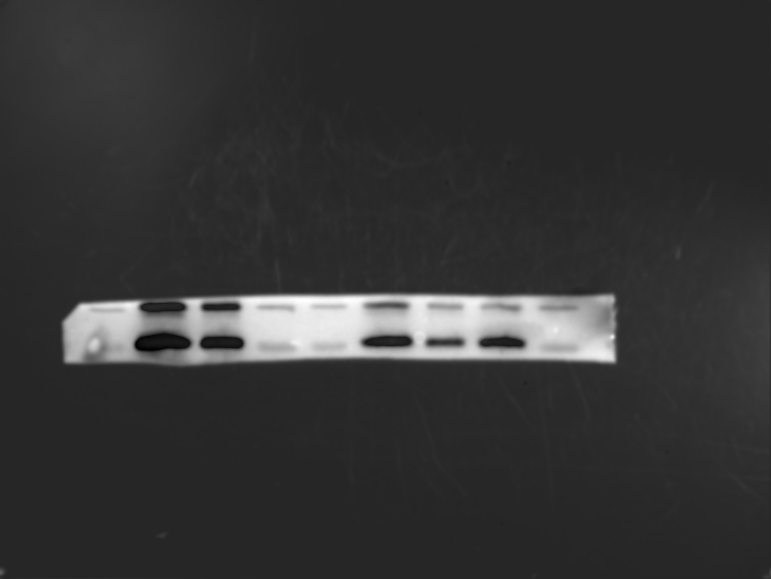

Supplement: Supplementary file 5 — Additional file 5. [file 13075_2023_3221_MOESM5_ESM.zip › Supplement4/Figure5F/CRB3.tif]

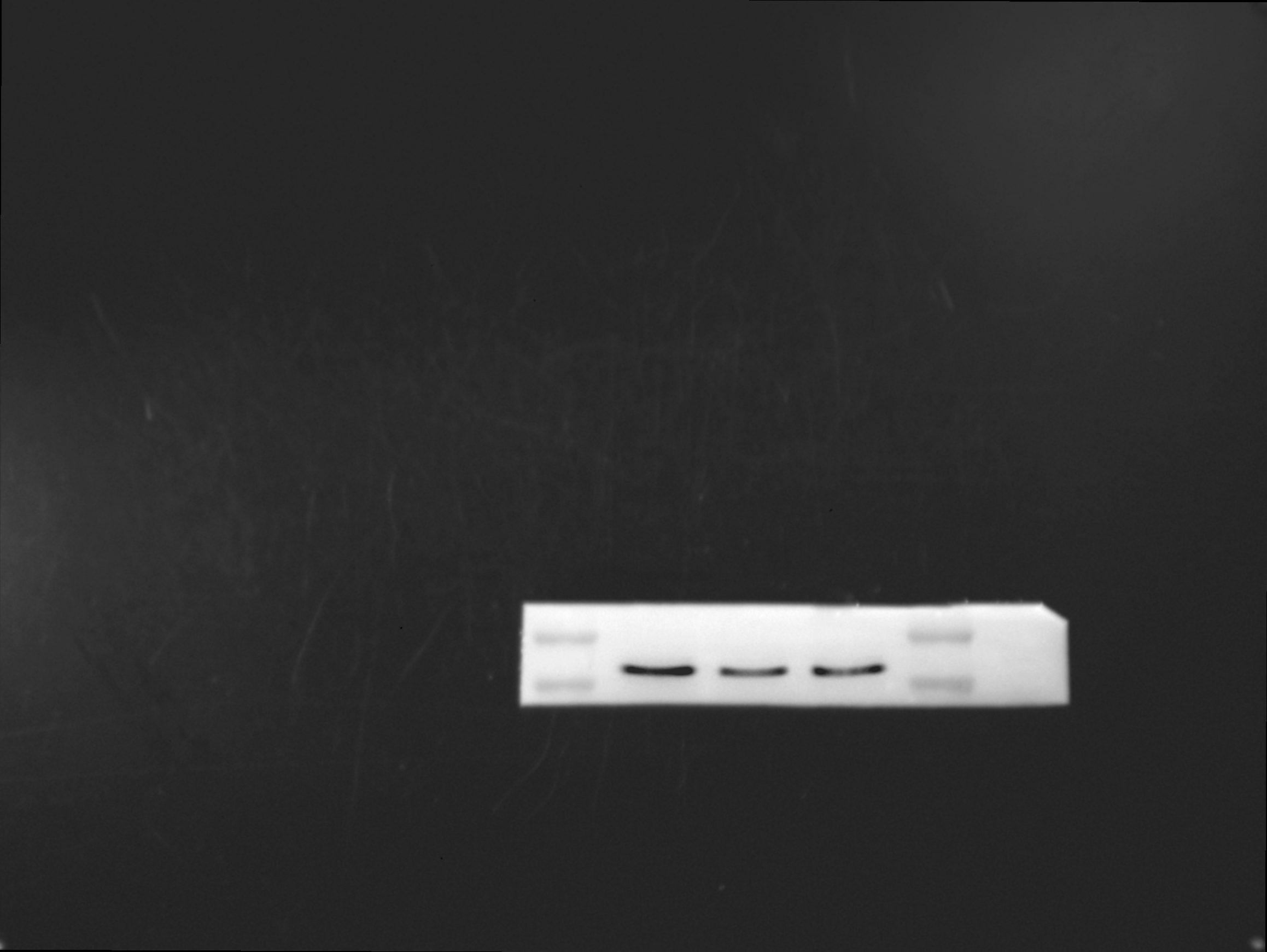

Supplement: Supplementary file 5 — Additional file 5. [file 13075_2023_3221_MOESM5_ESM.zip › Supplement4/Figure5F/FBP1.tif]

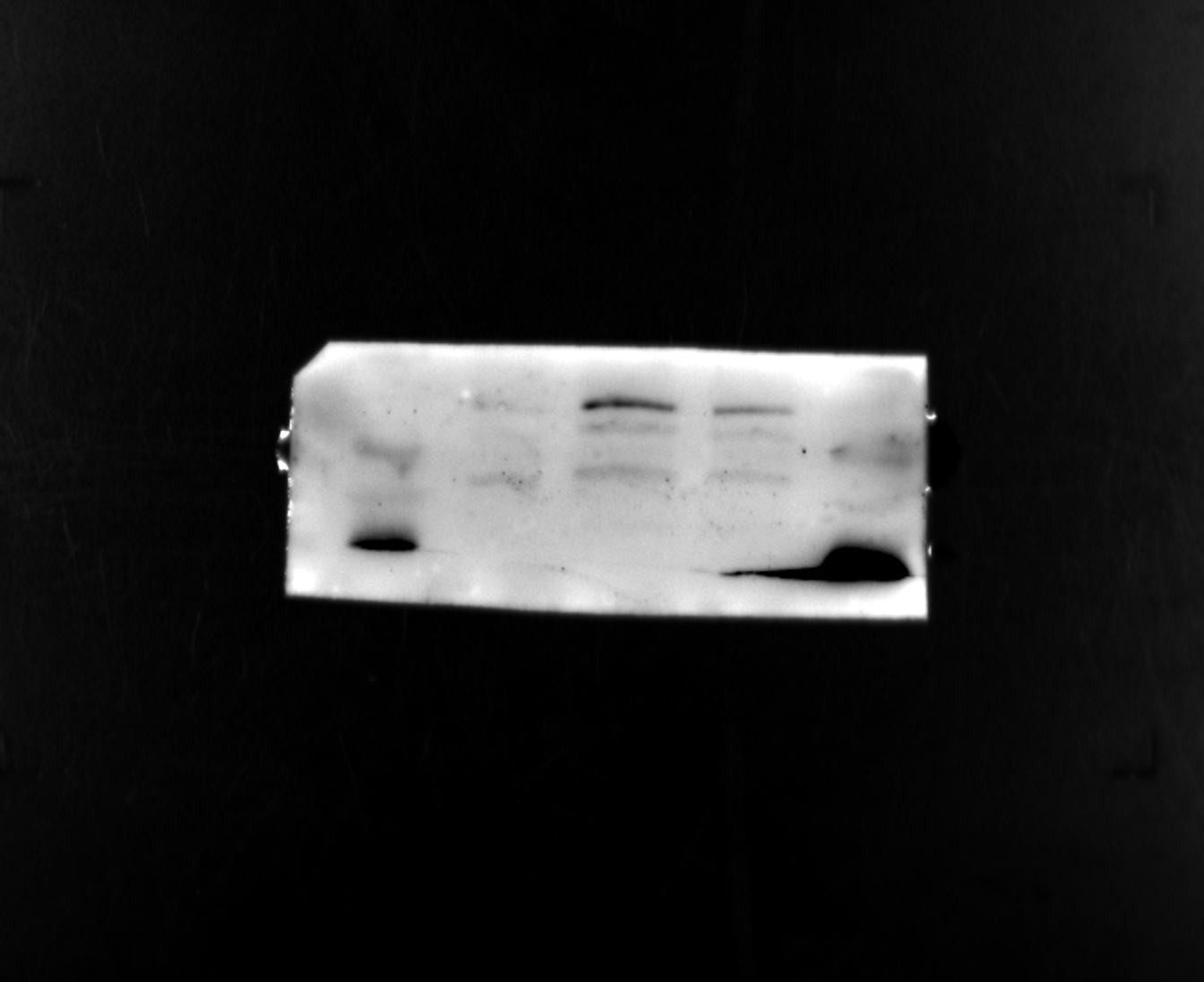

Supplement: Supplementary file 5 — Additional file 5. [file 13075_2023_3221_MOESM5_ESM.zip › Supplement4/Figure5F/P16.Tif]

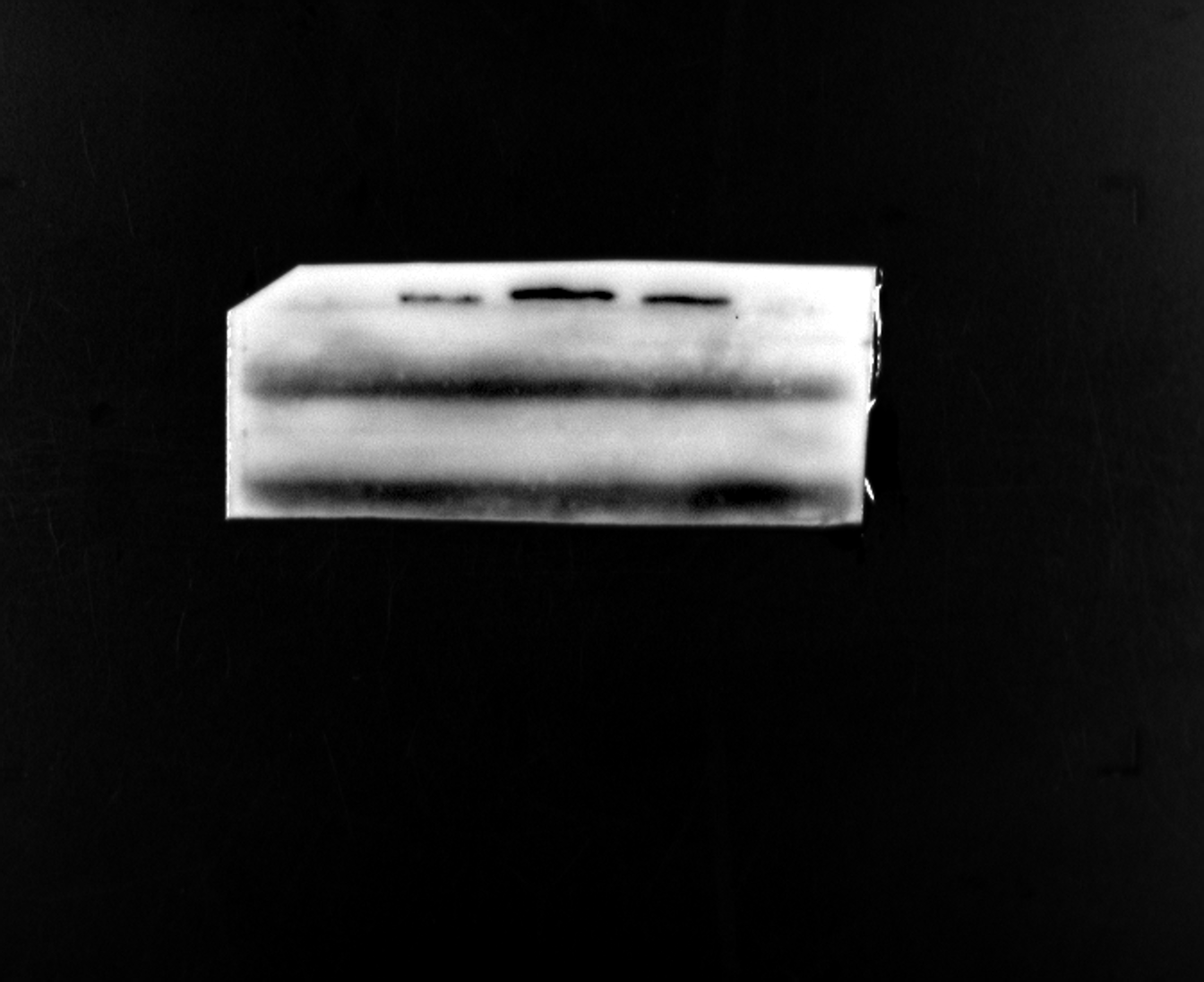

Supplement: Supplementary file 5 — Additional file 5. [file 13075_2023_3221_MOESM5_ESM.zip › Supplement4/Figure5F/p21.Tif]

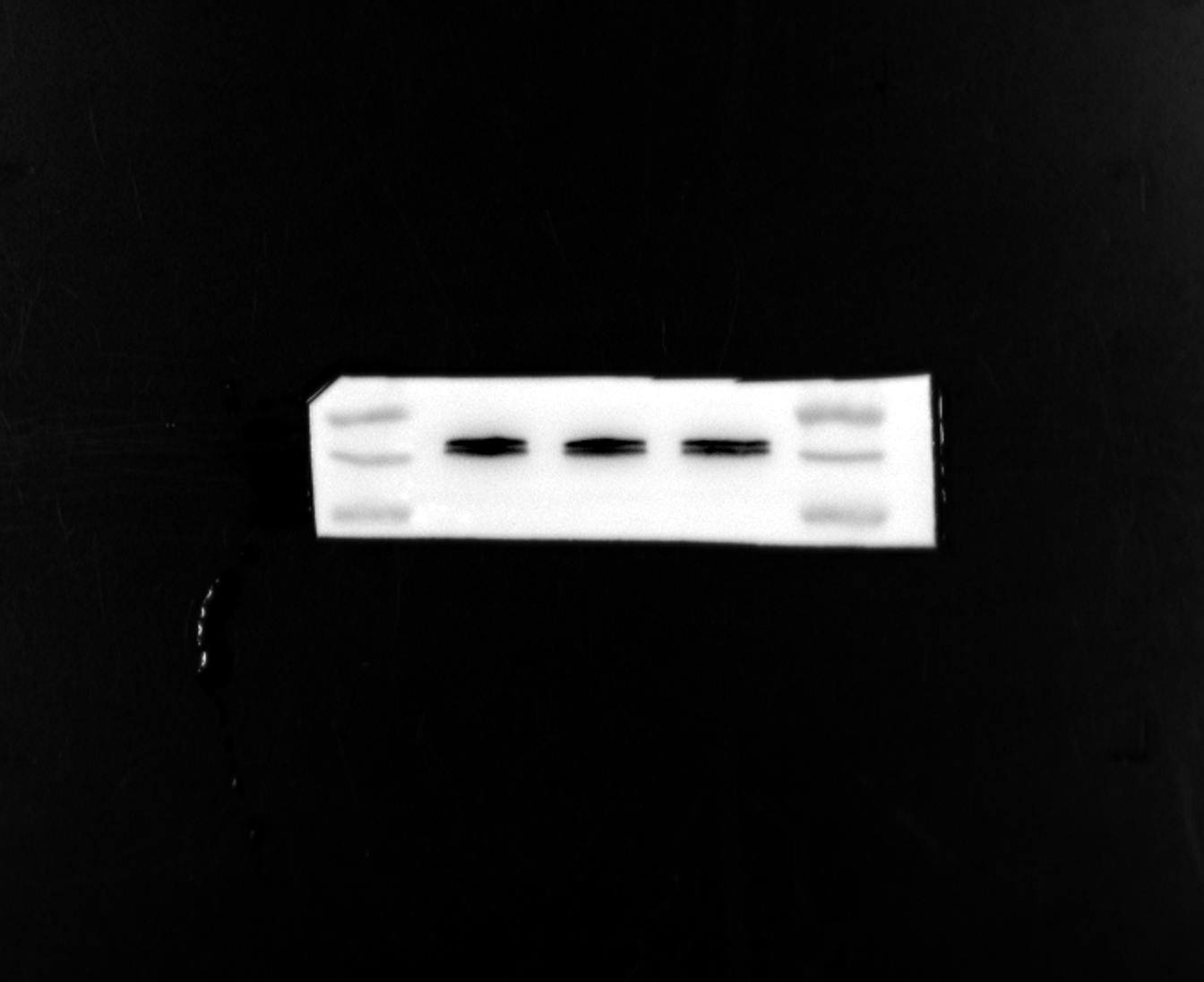

Supplement: Supplementary file 5 — Additional file 5. [file 13075_2023_3221_MOESM5_ESM.zip › Supplement4/Figure5F/β-actin(3).Tif]
